# Supplementary material for: Scalable high yield exfoliation for monolayer nanosheets
Source: Nat Commun. 2023 Jan 16;14:236. doi: 10.1038/s41467-022-35569-8 (PMC9842657; doi:10.1038/s41467-022-35569-8)
Supplement: Supplementary file 1 — Supplementary information [file 41467_2022_35569_MOESM1_ESM.pdf]

Supplementary information

Scalable High Yield Exfoliation for Monolayer Nanosheets

Zhuyuan Wang[1], Xue Yan[2], Qinfu Hou[1], Yue Liu[1], Xiangkang Zeng[3], Yuan Kang[1], Wang Zhao[1], Xuefeng Li[1], Shi Yuan[1], Ruosang Qiu[1], Md Hemayet Uddin[4], Ruoxin Wang[1], Yun Xia[1], Meipeng Jian[1], Yan Kang[5], Li Gao[6], Songmiao Liang[5], Jefferson Zhe Liu[2], Huanting Wang[1], Xiwang Zhang[1,3]*

[1] Department of Chemical and Biological Engineering, Monash University, Clayton, VIC, 3800, Australia

[2] Department of Mechanical Engineering, The University of Melbourne, Parkville, VIC 3010, Australia

[3] UQ Dow Centre for Sustainable Engineering Innovation, School of Chemical Engineering, The University of Queensland, St Lucia, Queensland, 4072, Australia

[4] Melbourne Centre for Nanofabrication, 151 Wellington Road, Clayton 3168, Australia

[5] Vontron Membrane Technology Co. Ltd., No. 1518 Liyang Road, Guiyang, Guizhou 550014, P. R. China

[6] South East Water Corporation, PO Box 2268, Seaford, Victoria 3198, Australia

* Email: xiwang.zhang@uq.edu.au

Supplementary Contents

Supplementary Section 1. Experiment 4

Supplementary 1.1 Materials 4

Supplementary 1.2 Processing parameters 4

Supplementary 1.3 Preparation of bulk materials 5

Supplementary 1.4 Preliminary screening 6

Supplementary 1.5 Rinsing 7

Supplementary 1.6 Preparation of powder and liquid dispersions 8

Supplementary 1.7 Characterizations 8

Supplementary 1.8 Applications 10

Supplementary Section 2. Exfoliation performance 12

Supplementary 2.1 Apparent height of graphene nanosheets measured by AFM 12

Supplementary 2.2 Lateral size tunability 14

Supplementary 2.3 Further evidence on monolayer graphene 16

Supplementary 2.4 Analysis on large multilayers 18

Supplementary 2.5 Comparison with other non-chemistry synthesis methods 19

Supplementary Section 3. Practicability 21

Supplementary 3.1 Sale-up study 21

Supplementary 3.2 Dispersibility of graphene nanosheets in different solvents 22

Supplementary 3.3 Drying for storage and Re-dispersion 23

Supplementary Section 4. Residual PEI 25

Supplementary 4.1 Concentration of residual PEI 25

Supplementary 4.2 Removal of residual PEI 26

Supplementary 4.3 Conductivity of graphene nanosheets 28

Supplementary 4.4 Zeta potential of synthesized nanosheets 29

Supplementary 4.5 Ion transport of HCl-treated membranes 31

Supplementary Section 5. Exfoliation mechanism 32

Supplementary 5.1 Graphene at intermediate stage 32

Supplementary 5.2 DEM simulation 33

Supplementary 5.2.1 Governing equations 33

Supplementary 5.2.2 Parameters for the DEM simulation 34

Supplementary 5.2.3 Results and discussion of DEM simulation 34

Supplementary 5.3 Surface morphology characterization of grinding balls 37

Supplementary 5.4 Effect of PEI:graphite ratio 37

Supplementary 5.5 Density Functional Theory (DFT) calculation 38

Supplementary 5.5.1 Calculation methods 38

Supplementary 5.5.2 Results and discussion of DFT 39

Supplementary 5.6 Effect of viscosity of PEI mixtures 42
Supplementary 5.7 Calculation of viscosity threshold for obtaining monolayer graphene 43
Supplementary Section 6. Universality of sticky mechanical exfoliation 44
Supplementary 6.1 Other layered crystals 44
Supplementary 6.1.1 Exfoliation of TAPB-PDA COF 44
Supplementary 6.1.2 Exfoliation of ZIF-L 45
Supplementary 6.1.3 Exfoliation of g-C_3N_4 47
Supplementary 6.2 Exfoliation of BN 48
Supplementary 6.3 Comparison with existing methods 52

Supplementary Section 1. Experiment

Supplementary 1.1 Materials

Polyethyleneimine (PEI), Polyethylene glycol 400 (PEG-400), Graphite (product number 496596, powder, <45 micron, >99.99%), Hexagonal Boron Nitride (h-BN) (product number 255475, powder, ~1μm), Ethanol, Dimethylacetamide (DMAc), Acetone, N-Methyl-2-pyrrolidone (NMP), Tetrahydrofuran (THF), Isopropanol (IPA), Dimethylformamide (DMF), $Zn(NO_3)_2 \cdot 6H_2O$ (98%), 2-methylimidazole (Hmim) (99%), Terephthalaldehyde, 1,4-dioxane, and mesitylene were purchased form Sigma-Aldrich and used as received. 1,3,5-tris(4-aminophenyl) benzene was provided by Tokyo Chemical Industry (TCI) chemicals and used as received.

Supplementary 1.2 Processing parameters

Supplementary Table 1.1 Viscosity of PEI mixture at two temperatures averaged from three measurements

Number	Mixing ratio (low-viscosity PEI : high viscosity-PEI)	Viscosity (mPa·s), at 20 °C	Viscosity (mPa·s), at 50 °C
Vis-1	1 : 0	1,508	244
Vis-2	1 : 0.5	7,235	1,068
Vis-3	1 : 1	16,858	2,364
Vis-4	0.5 : 1	33,716	5,380
Vis-5	0 : 1	150,882	28,717

Note: Low-viscosity PEI represents PEI with low-molecular weight with average Mn at ~600 by GPC. High-viscosity PEI represents PEI with high-molecular weight with average Mn at ~10,000 by GPC.

Supplementary Table 1.2 Basic milling parameters for different layered crystals

Layered crystals	PEI mixture	PEI loading amount (weight ratio to layered materials)	Milling speed (Revolution, rpm)	Milling time (hours)
Graphite	Vis-1 to Vis-5	4	500	5-15
Boron nitride-1	Vis-5	2	500	15
Boron nitride-2	Vis-5	2	500	25
Boron nitride-3	Vis-5	2	600	15

Carbon nitride	Vis-5	4	500	15
ZIF-L	Vis-5	4	500	5
TAPB-PDA COF	Vis-5	4	500	15

Note: The amount of added PEI mixture has direct impacts on the exfoliation of layered materials and the protection of exfoliated nanosheets. The impacts are discussed in detail in section 5.

Supplementary 1.3 Preparation of bulk materials

Graphitic Carbon Nitride: Bulk graphitic carbon nitride (g-C_3N_4) was prepared via a two-step calcination method[1]. Briefly, urea powder (10 g) was added to a crucible (100 mL capacity) with a cover (sealed with aluminum foil) and calcined at 550 °C for 2 h with a heating rate of 5 °C/min in a muffle oven under air condition. Then, the obtained faint yellow powder (1 g) was ground and filled into a crucible (same capacity) and calcined again under same condition. The final light-yellow powder was stored at room temperature before exfoliation experiments.

ZIF-L: Bulk ZIF-L powder was synthesized following the reported procedures[2]. Briefly, 0.59 g of $Zn(NO_3)_2 \cdot 6H_2O$ and 1.30 g of Hmim were dissolved in 40 mL Mill-Q water respectively. The zinc nitrate solution was then added into the Hmim solution under constant stirring. The reaction lasted for 4 h at room temperature. The product was collected by centrifugation (RCF 3823 g, 20 min) and washed by water and ethanol for two times. It is worthy to be mentioned that ZIF-L is not stable in aqueous environment without excessive Hmim although it was synthesized in water. Therefore, ethanol was chosen as the solvent for processing.

TAPB-PDA COF: COF powder (TAPB-PDA) was prepared according to a low temperature synthesis route catalyzed by metal triflates[3]. Briefly, Terephthalaldehyde (40 mg, 0.30 mmol) and 1,3,5-tris(4 aminophenyl)benzene (72 mg, 0.205 mmol) were added to a 25 mL scintillation vial in coupled with a 1,4-dioxane/mesitylene mixture (4:1 v/v, 16 mL). This mixture was first sonicated for 10 s and then heated to 70 °C for 5 minutes to ensure that all solids had dissolved, Scandium (III) trifluoromethanesulfonate (6 mg, 0.012 mmol) was added to the solution after temperature cooling back to room temperature, and it was then sonicated for 10 s immediately. The reaction solution was left without stirring for 2.5 h, and then a significant amount of brick red solid precipitate was obtained. The sediment was filtrated on a nylon membrane (pore size: 0.22 um, diameter 47 mm, Sterlitech, USA) and washed by the mixed solvent (used for synthesizing) 3 times. The as-prepared solid was further washed by methanol in a Soxhlet extractor for 12 h and freeze dried, a yellow TAPB-PDA COF powder was finally gained.

Supplementary 1.4 Preliminary screening

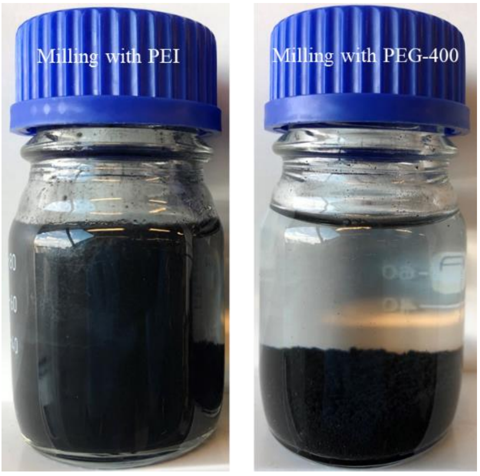

Supplementary Fig. 1.1. Photograph of water dispersions of graphite milling with PEI and PEG-400 for 15h. Milling conditions: Graphite=0.5 g, PEI/PEG-400=2g, Milling time=15 h, Milling speed=500 rpm.

Two kinds of polymer liquids were tested as exfoliation assistants in our preliminary experiments. After milling for 15h, graphite milling with PEI can be well dispersed in water, which is one of the important clues of effective exfoliation. Conversely, graphite milling with PEG-400 does not appear to be dispersible in water, which means an ultra-low exfoliation yield. The ineffectiveness of PEG-400 could be explained by the inefficient force transmission efficiency because of the low viscosity of PEG-400 (only at around 122 mPa·s, measured at 20 °C), or it could be its low adsorption energy on the graphene surface. However, our DFT calculation results found the adsorption energy of both PEI and PEG on graphene are greater than the interlayer binding energy of graphite (Supplementary Table 5.1). The key difference between PEI and PEG on their properties are their viscosity, suggesting the crucial role of high viscosity in this sticky exfoliation strategy.

Supplementary 1.5 Rinsing

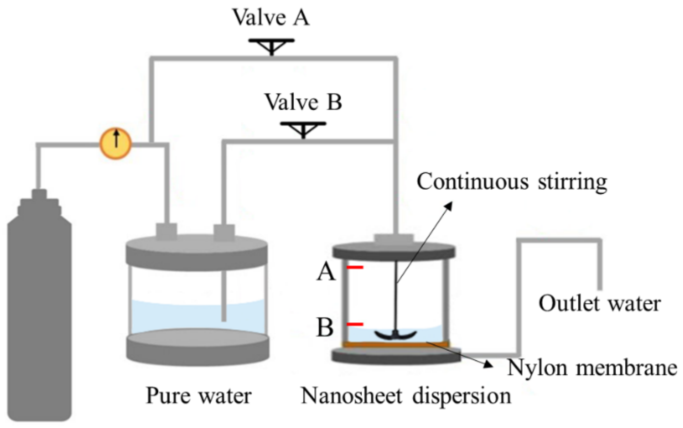

Supplementary Fig. 1.2. Schematic of the rinsing set-up.

Removing additives from nanosheet dispersion is usually time-consuming and lab-intensive in 2D materials synthesis. Vacuum-assisted filtration is one of the commonly adopted methods. However, it is only suitable for rinsing a small amount of 2D materials. This is because of the fact that 2D materials are prone to parallelly stacking with each other under the directional water flow and form a laminar film on filter[4]. This film not only dramatically reduces the rinsing efficiency but also blocks the additives from being washed out. Another method is dialysis. It, however, requires a large amount of water and is time-consuming as well.

In this work, we developed a rinsing process by taking advantage of membrane filtration. The membrane filtration set-up was designed to avoid solutes accumulating on the membrane surface so as to minimize concentration polarization. Briefly, 10 mL nanosheet dispersion was filled into a stainless-steel dead-end cell (HP 4750, Sterlitech, USA) under constant stirring to avoid agglomeration or forming a fine filtration cake. A nylon ultrafiltration membrane (pore size: 0.22 um, diameter 47 mm, Sterlitech, USA) was loaded at the bottom of the dead-end cell. The cell was connected with a 5 L tank with feed milli-Q water. They were connected with a gas cylinder with compressed N_2. The rinsing process starts with charging the water into the cell (by opening valve B and closing valve A in Supplementary Fig 1.2) until the liquid level in cell reaches marker A (around 100 mL). Then by closing valve B and opening valve A (Supplementary Fig. 1.2), water with dissolved PEI molecules will flow out of the cell driven by a pressure gradient, while nanosheets are blocked inside the cell by the membranes. This process lasted until the water level in cell reached mark B (around 10 mL) before filling cell with water to mark A again. The rinsing needed to be repeated several times to adequately remove free PEI from nanosheet dispersion. It should be noted that for boron nitride and carbon nitride, we washed the nanosheets with 0.1M HCl three times followed by water to partially defunctionalize the nanosheets in order to ensure a better resolution for

AFM characterization (Supplementary Section 4.2). Ethanol was chosen as the solvent for the rinsing of ZIF-L instead of water concerning its poor stability in water[5]. A UV-Vis spectrophotometer (UV-2600, Shimadzu) was adopted to detect the concentration of PEI in outlet water. In this work, we continued the resining step until the PEI concentration in outlet water is less than less than 0.0001 mg/mL. The rinsing method effectively mitigates nanosheet agglomeration, avoids the formation of laminar membranes, and achieves high removal of free PEI molecules.

Supplementary 1.6 Preparation of powder and liquid dispersions

After finishing rinsing, a certain amount of water was added to the cell to ensure the liquid volume was around 20 mL. The cell was then left stirring for 30 min. Although under continuous stirring, a filter cake still formed on the membrane during rinsing process. Therefore, the dispersion was then decanted to a beaker, the nylon membrane with a filter cake was taken out and put into a beaker as well. The beaker with dispersion was then subject to an ultrasonication for 30 min using a Unisonics FXP12M sonic bath (40 kHz, 100 W). Subsequently, the dispersion was centrifugated at RCF of 236 g (Sigma 2-16P) for 20 min to remove thick flakes. The supernatant was transferred to give a final dispersion product. To evaluate the exfoliation yield, 5 mL of dispersion was filtrated onto a pre-weighed ultrafiltration membrane and the concentration was calculated according to the weight difference after drying the membrane for 24 h in an oven with temperature at 60 °C. The apparent yield was then determined according to the dispersion concentration. Noted that the apparent yield was calculated based on the weight of as-prepared nanosheets, which means that small amount of residual PEI on the nanosheets was also included. The actual yield is a little bit lower, which can be calculated by subtracting the weight of residual PEI (in the range of 6.26 wt.% to 12.43 wt.%) according to the result of elemental analysis (Supplementary Section 4.1). The rinsed dispersion was freeze-dried (FreeZone 2.5 liters, Labconco Corporation, USA) to obtain powder samples for storage.

Supplementary 1.7 Characterizations

The thickness and lateral size of the exfoliated nanosheets were characterized by atomic force microscopy (Bruker Dimension Icon). To prepare the samples, the rinsed water dispersion of graphene nanosheets was diluted with ethanol to a concentration of 5 μg/mL after sufficiently washing out free PEI molecules. The graphene/ethanol solution was then sonicated for 1 h using a Unisonics FXP12M sonic bath (40 kHz, 100 W). Freshly exfoliated mica (12.5 mm, grade V-1, ProSicTech) plates were chosen as the substrates and the exposed new surface was washed with ethanol after exfoliation using scotch tape. Before the ethanol on the mica totally dries, 3 μL of the diluted dispersion was dropped onto it using a pipette, and then carefully shook the mica in order to

ensure the ethanol dispersion is evenly distributed on the surface. The mica with graphene nanosheets on top was then left in a 45 °C vacuum oven overnight before AFM characterization. Tapping mode was chosen for the analysis, and a relatively hard cantilever (TESPA-V2, BRUKER) was applied accordingly. The converged beam electric diffraction (CBED), selected area electron diffraction (SAED), transmission electron microscopy (TEM), high-resolution transmission electron microscopy (HRTEM), and scanning transmission electron microscopy (STEM) were obtained using a FEI Tecnai G2 F20 S-TWIN transmission electron microscope. The TEM samples were prepared by dipping a holey carbon film (300 mesh) into the graphene dispersion of 5 μg/mL and was dried under an ambient environment. The carbon state of the graphene samples was investigated by a Renishaw via Raman Microscope (Chameleon He−Ne laser generator with λ = 532 nm). The samples for Raman characterization were prepared following the same procedures as that of AFM using graphene fabricated by15-h milling. X-ray photoelectron spectroscopy (XPS) was performed on a Thermo Scientific Nexsa Surface Analysis System equipped with a hemispherical analyzer using powder samples. Scanning electron microscope (SEM) was operated at 3 kV with a working distance of 5mm (Nova NanoSEM 450, FEI, U.S.A.). All samples were coated with iridium (1.5 to 2 nm thick) prior to characterization. Fourier-transform infrared spectra were performed by the spectrometer (FT-IR/FIR, Frontier, PerkinElmer) using powder samples. Zeta potential of the nanosheet water suspensions was measured at a concentration of 0.05 mg/mL (Malvern, Zetasizer Nano Z). X-ray diffraction (XRD) analysis was performed from 5-100 °C by a diffractometer equipped with Cu Kα radiation (Miniflex 600 X-ray diffractometer, Rigaku, Japan; D2 PHASER powder diffractometer, Bruker, Germany) using freeze-dried powder samples. TGA was carried out from 50 to 900 °C with the temperature rising rate of 10 °C/min under continuous nitrogen flow (PerkinElmer TGA 8000). Viscosity of PEI was measured by a viscometer (ROTAVISC Io-vi, IKA, Germany). The elemental content (C, N, and H) of samples was performed on an Elemental Analyzer (FlashSmart, Thermo Scientific). The pure chemical standard 2, 5-Bis (5-tert-butyl-2-benzo-oxazol-2-yl) thiophene (BBOT, Elemental Microanalysis) was used to establish standard curves for elemental analysis. All samples were put in a Tin capsule and analysed three times. The sheet resistance of graphene films was measured by Keithley 2604B SourceMeter (Tektronix, USA) equipped with a 4-point probe. The surface roughness of 5-mm grinding balls was characterized by Bruker optical profilometer (Contour GTI 3D optical profiler, USA).

Supplementary 1.8 Applications

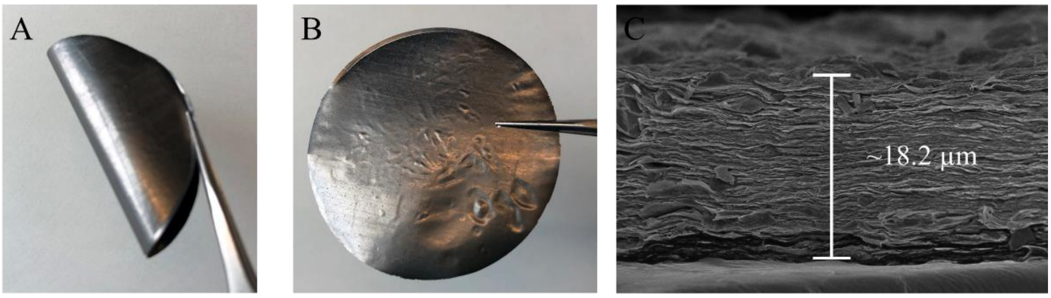

Supplementary Fig. 1.3. The conductive graphene films. (A) (B) Photographs of flexible graphene film. (C) Thickness of the graphene film indicated by SEM cross-section imaging.

Fabrication of conductive membranes: Graphene nanosheet (synthesized under the conditions: PEI=Vis-5; milling time=10h) dispersions in water were diluted to a concentration of 0.01 mg/mL and then filtrated onto a PES membrane (pore size: 0.03 μm, diameter 47 mm, Sterlitech, USA) at different loading density to obtain graphene films with a wide range of thickness. After filtration, the graphene/PES films were dried under ambient temperature for 5h. For thin membranes, the conductivity was measured directly with PES substrates. For thick ones, flexible free-standing graphene films were fabricated by following a freeze-and-sublimation transfer strategy[6]. In brief, the graphene/PES film was placed onto a clean glass substrate with the graphene side facing the glass. Several drops of water were then added on the backside of the film to wet the film for 10 min. The film with the glass substrate was kept in a freezer (-20 °C) until the graphene film automatedly separated with the PES substrate and become a free-standing film. The graphene films show a flexible nature and decent mechanical strength (Supplementary Fig. 1.3).

Residual PEI molecules on graphene nanosheets were removed partially and totally to study their impacts on conductivity. For HCl-treated graphene films, 100-mL 0.1 M HCl was filtrated immediately after finishing the filtration of graphene dispersion, which was then followed by repeated filtration of 100 mL milli-Q water 5 times. This process can remove around 45% of residual PEI according to the results in supplementary Section 4.2. In an effort to completely remove residual PEI, the obtained thick free-standing graphene film was subject to a heat treatment described in supplementary Section 4.2. Interestingly, the graphene film can maintain its original shape without breaking into small pieces and the weight loss after the heat treatment is at around 10%, which is close to the percentage of residual PEI.

Fabrication of ion separation membranes: Graphene membranes with laminar ion channels were fabricated by vacuum filtration (Welch, 2511 WOB-L Pump) of diluted graphene dispersion (~1 mg/L) using PES ultrafiltration membranes (pore size: 0.03 μm, diameter 47 mm, Sterlitech, USA).

Graphene nanosheets obtained with PEI Vis-5 mixture after 10 h milling were used considering its reasonable lateral size and high monolayer percentage. The thickness of the laminar graphene membranes can be controlled by changing the loading amount of graphene solution. For demonstration, the thickness of the graphene membranes in this work was controlled at around 500 nm (Supplementary Figs. 1.4A-B). The graphene membranes were put into a vacuum desiccator immediately after filtration process was completed and stored overnight before testing. The HCl-treated membranes were fabricated following the same procedures except washing three time using 100-mL 0.1 M HCl after finishing the filtration of graphene solution.

Ion transport experiments: The ion transport behavior in laminar graphene membranes was studied in a concentration-gradient-driven ion diffusion process using a homemade two-compartment PTFE diffusion cell. The graphene membrane with PES substrate was mounted between two compartments with a hole of 8 mm in diameter. 15 mL of salt solutions ($MgCl_2$, $CaCl_2$, KCl, NaCl) with a concentration of 0.5 M and the same amount of milli-Q water was simultaneously added to the left and right side, respectively. To minimize concentration polarization, the graphene layer side was placed facing salt solutions while the PES substrate faced DI water. Constant stirring was applied throughout the test. The salt concentration in the permeation side was tested every 10 min by a conductivity meter (labCHEM-CP). The experiments were conducted at room temperature (20°± 1°C). Ion permeation rate (J_i) was calculated as follows.

$$J_i = \frac{C_t V}{At} \qquad (1.1)$$

where J_i is the ion permeation rate. V is the total volume of permeate solution (15 mL). C_t is the salt concentration in permeated solution. A is the effective area of graphene membrane (50.27 mm^2).

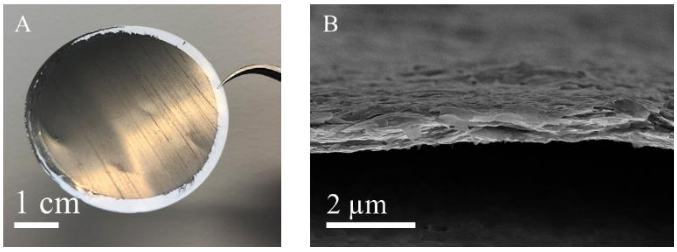

Supplementary Fig. 1.4. The larimar graphene membrane. (A) Digital photo of the fabricated graphene laminar membrane. (B) Cross-section morphology of the laminar graphene membrane imaged by SEM.

Supplementary Section 2. Exfoliation performance

Supplementary 2.1 Apparent height of graphene nanosheets measured by AFM

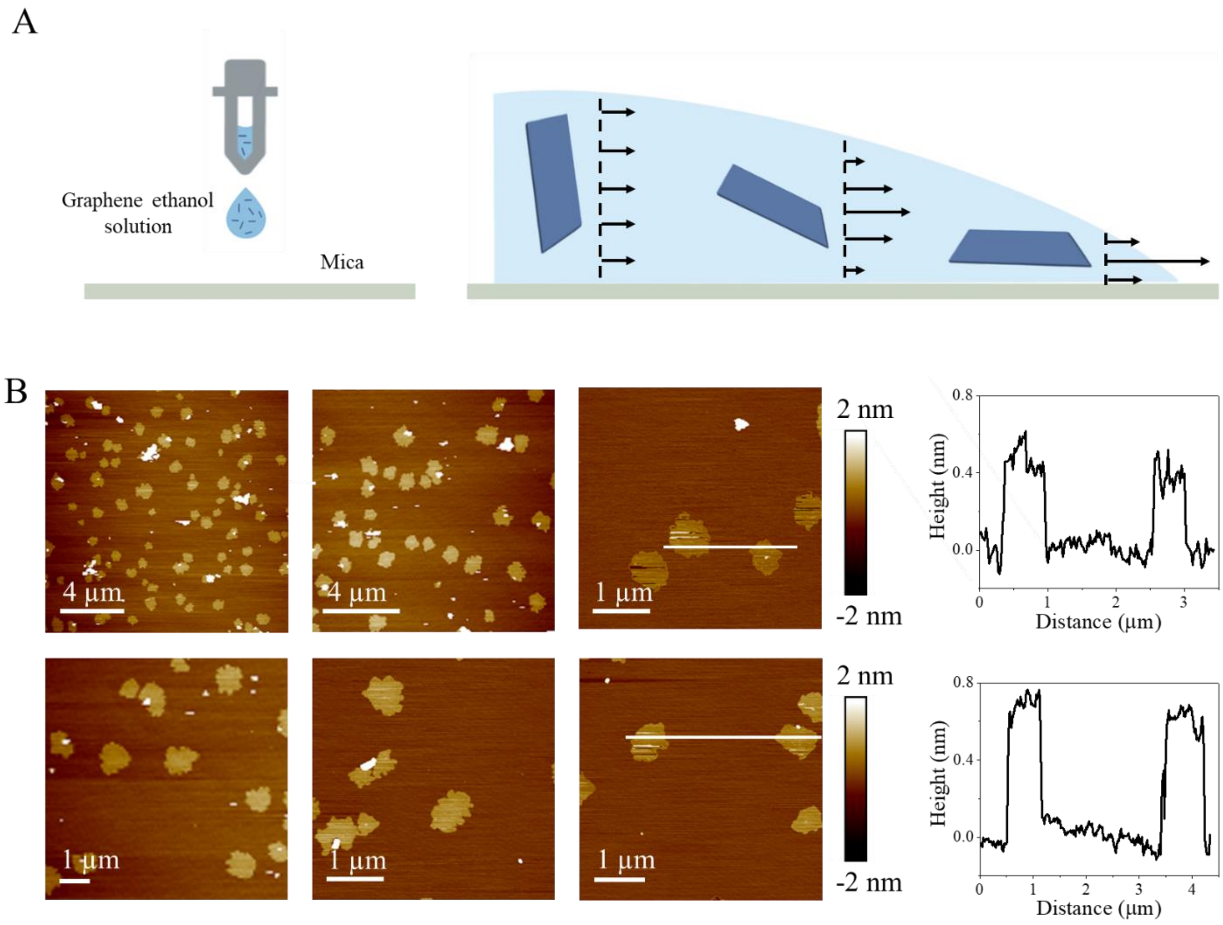

Supplementary Fig. 2.1. AFM characterization of graphene nanosheets. (A) Schematic of graphene nanosheet sample preparation for AFM characterization. (B) Selected AFM images for statistical analysis of graphene (PEI Vis-5, milling time 10h). The right column is the corresponding height profiles along the lines in the images.

In order to investigate the layer number of exfoliated nanosheets, the as-prepared nanosheets were characterized by AFM to measure their apparent heights. Graphene solution was diluted with ethanol for sample preparation. Since ethanol can quickly spread on the ethanol pre-wetted surface of the mica, which generates a strong shear-flow-induced alignment of 2D nanosheets (Supplementary Fig. 2.1A)[7]. Slightly negative charged surface of mica can also help spread the nanosheets, sample folding and overlapping can thus be largely avoided to encourage a more accurate analysis of the lateral size and thickness distribution of graphene nanosheets. The mica with graphene nanosheets was then left in a 45 °C vacuum oven overnight to totally remove the ethanol.

198 pieces of nanosheets were analyzed by AFM imaging and 182 of them show heights around ~0.7 nm (selected AFM images shown as Supplementary Fig. 2.1B). Graphene nanosheets with a height of less than 1 nm under AFM characterization are generally recognized as monolayers in literatures[8].

In this work, although the heights of thin graphene nanosheets are a little higher than the theoretical thickness (0.335 nm), they are less than the thickness of bilayers graphene (0.74 nm)[9]. The higher thickness of graphene obtained here than theoretical thickness is fairly reasonable. This is because the residual PEI molecules (around 11.5 wt.%, Supplementary section 4.1) on graphene nanosheets can act as spacer between graphene and mica as well as between cantilever and graphene, contributing to the measured heights. According to our simulation results, single-layer graphene nanosheets adsorbed with one-layer PEI possess a thickness of 0.95 nm (Supplementary Fig. 5.11A). Furthermore, the monolayer nature of the as-prepared graphene nanosheets is mutually supported by their Raman spectra and diffraction patterns, which are discussed in Supplementary Section 2.3. By following these lines of reasoning, we concluded that 91.2% of the obtained nanosheets are monolayer graphene, supporting the excellent exfoliation efficiency of this sticky ball-milling method.

Supplementary 2.2 Lateral size tunability

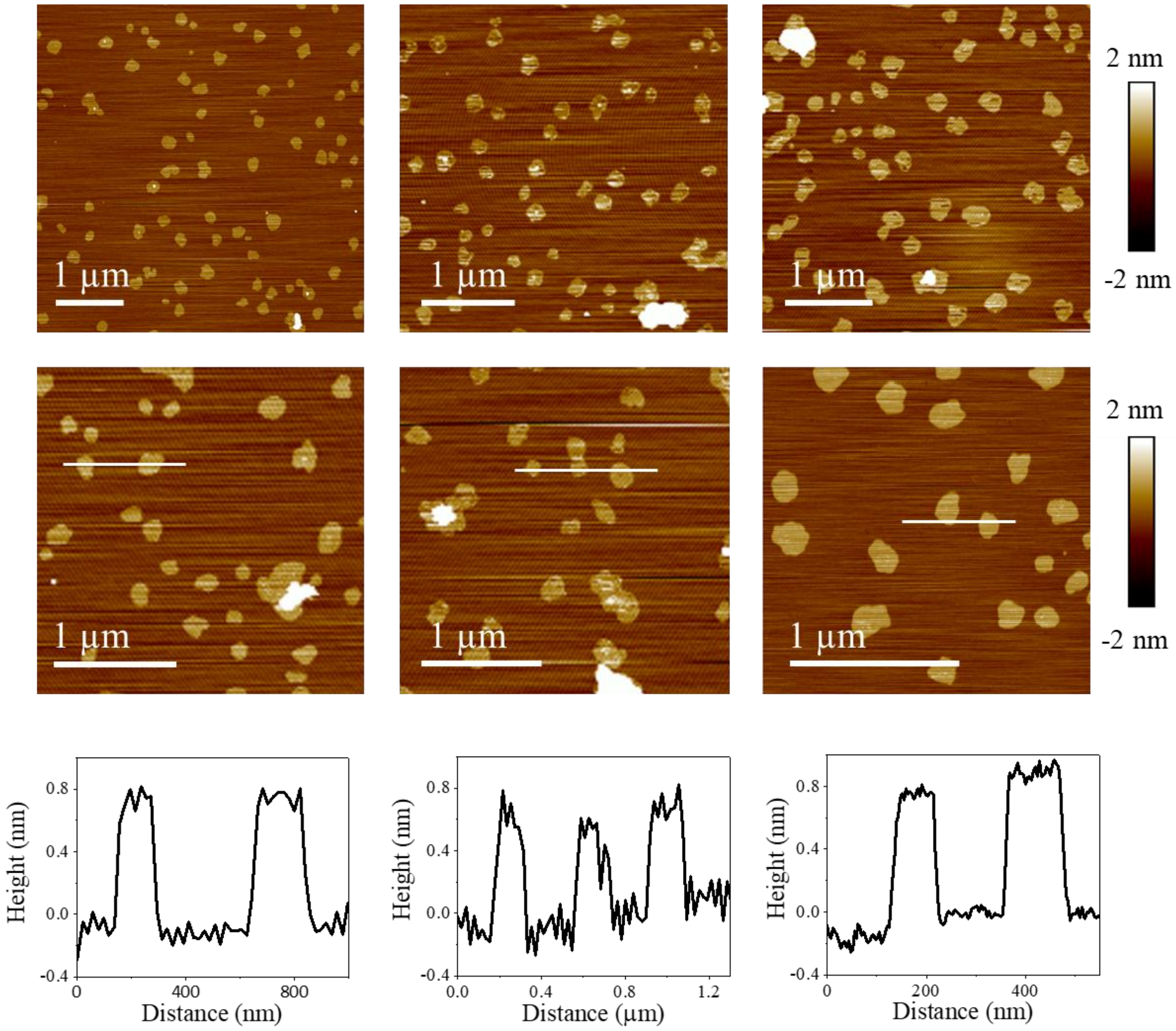

Supplementary Fig. 2.2. AFM images for statistical analysis of the as-prepared graphene nanosheets (PEI Vis-5, milling time 15h). All the images share the same height scale bar indicated in the right images. The bottom column is the corresponding height profiles along the lines in the above images.

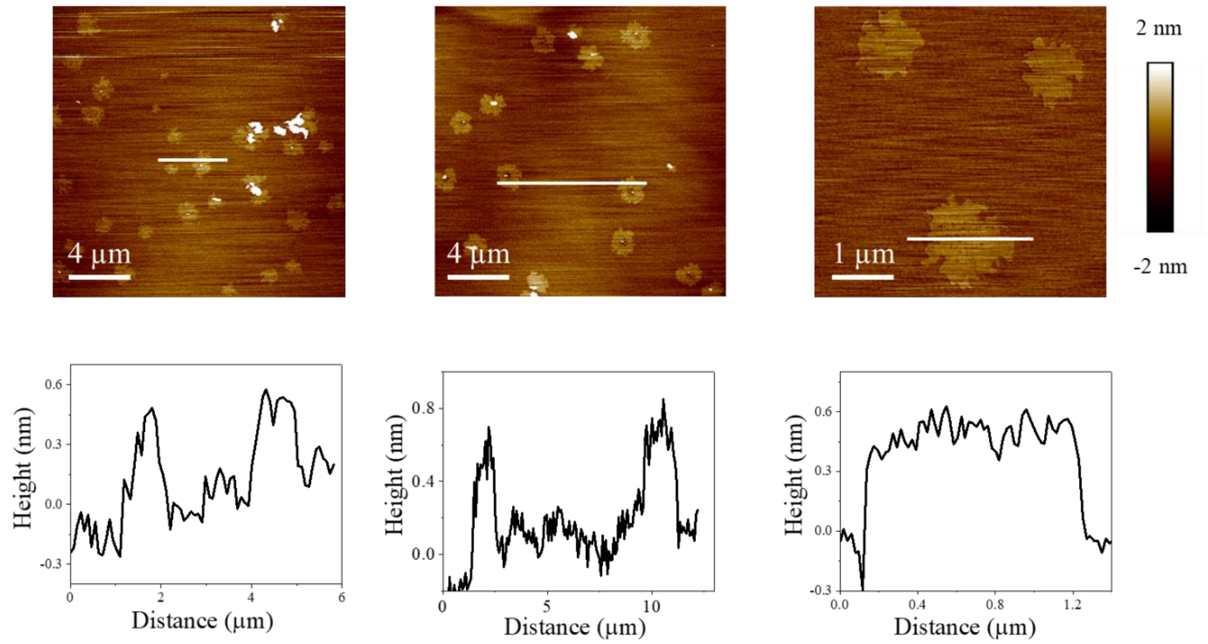

Supplementary Fig. 2.3. AFM images for statistical analysis of the as-prepared graphene (PEI Vis-5, milling time 5h). The right column is the corresponding height profiles along the white lines in the images.

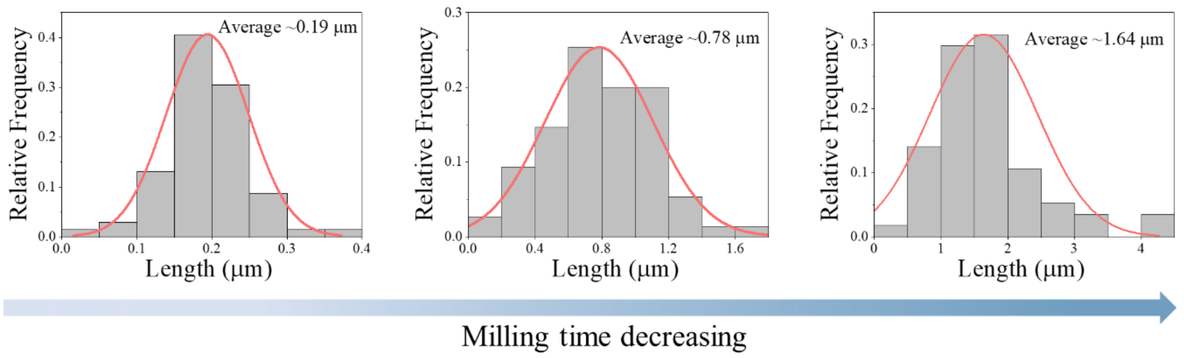

Supplementary Fig. 2.4. Lateral size distribution of the graphene nanosheets synthesized at different milling times. From left to right, graphene is exfoliated using PEI Vis-5 for 15 h, 10 h and 5 h, respectively. The lateral size distribution was analyzed based on AFM images, and at least 100 pieces of graphene nanosheets were counted for each of samples.

Lateral size is a crucial property for 2D materials, size tuneability is thus interesting for a wide range of applications. For example, small lateral size is beneficial for the interaction of nanosheets and molecules, therefore, nanosheets with their lateral size at tens to hundreds of nanometers are enough for use as catalysts[1]. However, for applications like using 2D materials as nanofiller or laminar membranes, large lateral size is ideal for matrix mechanical reinforcement and selective transport[4, 10]. In this sticky milling method, the lateral size of graphene nanosheets can be readily manipulated by varying processing time. As shown in Supplementary Figure 2.3, the average lateral size (the length of graphene nanosheet) decreases from 1.64 μm to 190 nm when extending the milling time from 5 h to 15 h. The lateral sizes of graphene nanosheets are mostly located in the range of 150 to 250 nm,

400 nm to 1 µm, and 1 µm to 2µm with response to the milling time of 15 h, 10 h, and 5 h, respectively. Interestingly, the obtained nanosheets are almost all in monolayer when milling for 15 h (only 11 pieces of multilayer sheets out of analyzed 522 pieces are not monolayered, corresponding to a monolayer percentage of 97.9%). Although the monolayer percentage decreases to ~76% as milling time is reduced to 5 h, the percentage is still much higher than most reported works as detailed in Supplementary Table 2.1.

The averaged apparent height of graphene nanosheets shows an increase from ~0.5 to ~0.8 nm along with the milling time extension from 5 h to 15 h. This might be the result that more PEI is bonded or adsorbed on graphene nanosheets with increase of milling time. The amount of PEI on as-prepared graphene nanosheets increases from 6.3 wt.% to 12.4 wt.% coupled with the milling time being extended from 5h to 15h (Supplementary Section 4.1). As a result, the gaps (nanosheets/mica, nanosheets/cantilever) get wider for the graphene nanosheets synthesized at longer milling time, leading to an increase in the apparent heights measured by AFM. What is interesting is that for graphene nanosheets exfoliated with 5-h milling, the apparent heights are around 0.5~0.6 nm. These heights are much lower than the theoretical thickness (0.67) and the observed thickness (0.74 nm) of bilayer graphene[9], further confirming that the graphene obtained in this work are indeed monolayers. Although for graphene obtained by 15-h milling, the heights (0.8-1 nm) overtake the thickness of bilayer graphene, we still believe that them are monolayers for the same reason. This is also under the assumption that the layer number of exfoliated nanosheets is not expected to increase with the extension of milling time. As we have confirmed that graphene nanosheets exfoliated by 5h and 10 h are mainly composed of monolayers, it is reasonable to take nanosheets obtained by 15-h milling are monolayers as well.

Supplementary 2.3 Further evidence on monolayer graphene

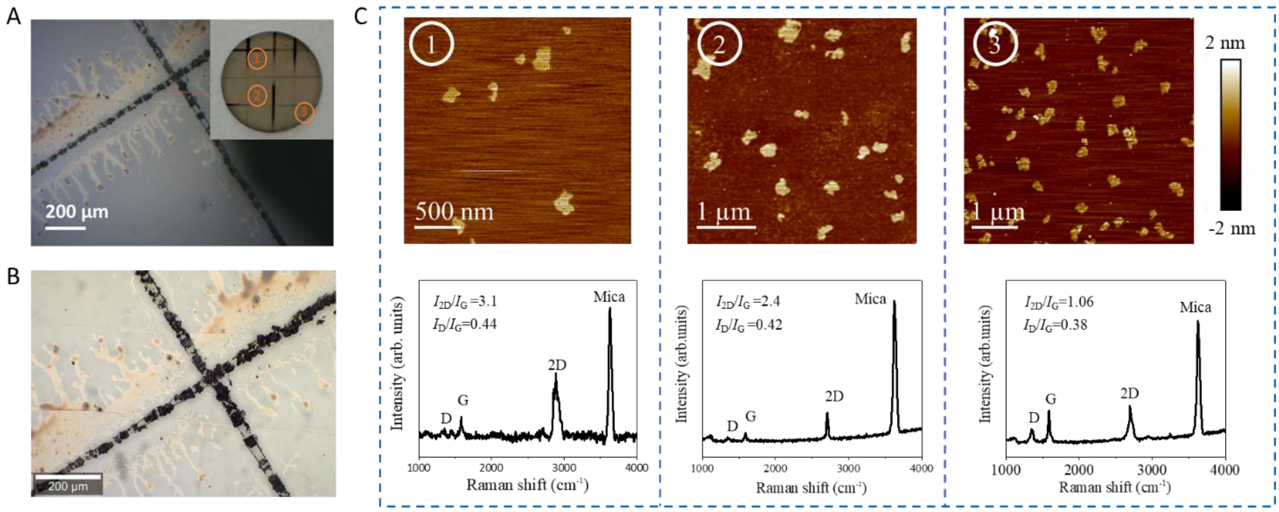

Supplementary Fig. 2.5. Raman characterization of graphene nanosheets. Point marker spotted by the optical microscope of AFM (A) and Raman device (B). Inset image in A is the photograph of the Raman sample with 9-point markers. (C) AFM images and Raman spectra obtained from the points indicated in A. Experimental conditions: integration time 1s, laser: 532-nm, accumulation times: 50 for position 1, 100 for position 2 and 3.

To provide further evidence for validating that the small discs spotted by AFM are graphene nanosheets, we tried to correlate the AFM morphology imaging with Raman structure mapping. Graphene fabricated from 15-h milling was deposited on mica following the same procedures of AFM sample preparation and used for demonstration. Since our nanosheets were not easily observed by Raman and AFM optical microscope, we manually marked our sample with 9 points to help us locate the same area in two separated characterization devices. The AFM characterization was firstly performed to find nanosheets around these points. As shown in the Supplementary Figure 2.5C, small discs were found in the area 1, 2, 3 with their thickness at around 1 nm and lateral size around 200 nm similar to what we found previously from AFM (Supplementary Fig. 2.5A). Once nanosheets were spotted in the vicinity of one of these points, Raman spectroscopy was then utilized to generate spectra from the same area with the help of our point markers on mica (Supplementary Fig. 2.5B). Repletely accumulated Raman signals indicate that there are plenty of pieces of graphene in these areas with identifiable typical D, G, 2D Raman peaks of graphene. All Raman patterns feature a symmetrical 2D band, suggesting the high monolayer percentage[11, 12]. Besides, the intensity ratios of I_{2D}/I_G of these generated Raman patterns locate in the range of 1.06-3.1 (Supplementary Fig. 2.5C). Giving that the I_{2D}/I_G band ratio is lower than 1 for bilayer graphene, and the ratio decreases with the increase of layer number[12]. In this line of reasoning, we conclude that the sub-nm discs observed by AFM are monolayer graphene. Furthermore, the I_D/I_G band intensity, which is related to sp^3 carbon defects, is in the range of 0.3-0.5. Such a low I_D/I_G ratio originated from the quality lattice is much lower than that of graphene oxide (GO) and reduced GO (rGO) (~0.7-0.9)[13], but is comparable to high-speed mixing delivered vacancy-defect-free few-layer graphene (0.17-0.37)[14].

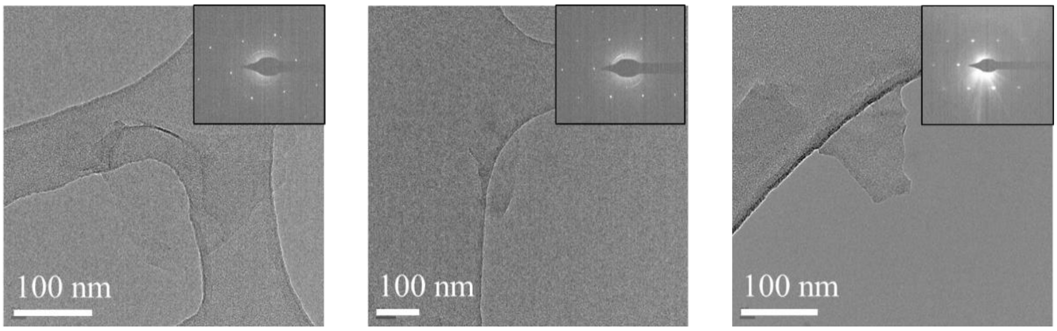

Supplementary Fig. 2.6. Low resolution TEM images of the graphene nanosheets (15-h milling). Insets are the SAED diffraction patterns of the spotted graphene.

Besides Raman spectroscopy, these small graphene discs were further observed by low resolution TEM and their diffraction patterns were obtained at the same time. The observed graphene nanosheets show a weak contrast to the carbon mesh background with their lateral size locates around 200nm, which is line with the result of AFM (Supplementary Fig. 2.6). Two types of diffraction patterns of the obtained graphene nanosheets were generated, with converged beam electric diffraction (CBED) was applied on part of ultra-large graphene nanosheets (Fig. 1e-g, main text) and selected area electron diffraction (SAED) was performed on the whole piece of small ones (Supplementary Fig. 2.6). These patterns exhibit a typical six-fold symmetry as expected from graphene or graphite. Besides, intensity scans along these diffraction patterns more intense inner spots ((0-110) and (-1010)) than outer ones ((1-210) and (-2110)), with an intensity ratio (*I* inner spots/*I* outer spots) >1, which is a characteristic fingerprint of monolayer graphene[11].

Supplementary 2.4 Analysis on large multilayers

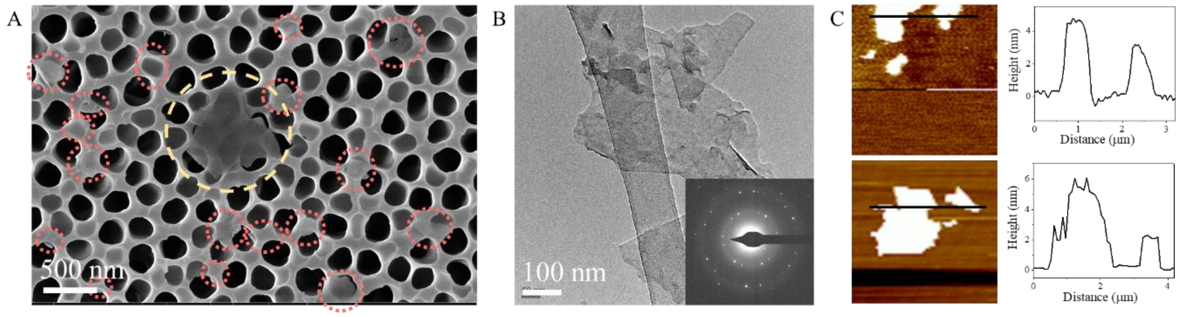

Supplementary Fig. 2.7. Characterizations of multilayer graphene. (A) SEM image of graphene nanosheets (15-h milling) deposited on a porous AAO disc. (B) TEM image of a multilayer graphene and its SAED diffraction pattern. (C) AFM topological image and height profile of multilayer graphene nanosheets.

When graphene nanosheets were deposited on a porous AAO disc, large nanosheets in the middle (yellow cycle) displaying a clear shape were observed. Although some small nanosheets can pass through the AAO disc that has a pore size similar to theirs, upon close observation, we still found many small nanosheets with round shapes and serrated edges. The morphologies of the small nanosheets agree with AFM observation (Supplementary Fig. 2.2), which are confirmed as monolayer graphene by Raman spectra and SEM diffraction patterns (Supplementary Figs. 2.5-2.6). By contrast, large nanosheets with sharp edges (Supplementary Fig. 2.7A) are possibly either the stack of several nanosheets with self-folding or multi-layers that have not been fully exfoliated, which are evidenced by TEM diffraction patterns and AFM height profiles (Supplementary Figs. 2.7B-C).

The morphology difference between monolayer and multilayer nanosheets could be the result of the in-plane breaking processes. Initial delamination and breaking start from structural deformation at mechanically vulnerable crystalline structural deformations on material surface, often named “kind

band striations"[15, 16, 17]. This crystalline structural breaking leads to incompletely exfoliated few-layer nanosheets with large sizes and sharp edges. The large nanosheets then undergo further in-plane breaking by collisions of protruding sharp ridges on the grinding ball surface (Fig. 3b), which provides sufficiently high compression to break graphene nanosheets at any point due to concentrated forces. Considering the highly random distribution of these ridges over the balls, the long edges of the nanosheets have a better chance to be trimmed than short edges during milling, as a result, smaller nanosheets that have undergone more times of breaking tend to be more likely to end up with higher roundness but with serrated edges.

Supplementary 2.5 Comparison with other non-chemistry synthesis methods

We have compared the sticky milling method with other scalable non-chemistry synthesis methods in terms of yield, lateral size, thickness, and monolayer percentage. The aspect ratio was calculated according to the averaged size and layer number reported in these references. However, some references only provided AFM measured heights, so we list the heights as provided instead of layer number. Hence, aspect ratios are given as nm/nm instead of nm/per layer for these cases. Few-layer graphene was obtained in most cases with the aspect ratio of graphene less than 500 nm/nm. Only two papers claimed that they got monodispersed single-layer graphene. One used salt-assisted ball milling to give graphene with a low yield at around 10% and small lateral size (~300 nm), and the other was Flash joule heating which was performed under ultrahigh temperature. Our method can produce graphene nanosheets with an actual yield of ~78.3 % and monolayer contents of over 90%. By applying short-time milling, the lateral size of obtained monolayer graphene can reach an average of 1640 nm (Supplementary Fig. 2.3).

Supplementary Table 2.1. Comparison with other non-chemistry methods

Methods	Size	Thickness	yield (dispersible nanosheets, %)	monolayer content (%)	Aspect ratio (nm/layer or nm)	Exfoliation medium	ref
Sonication (pre-activation by solvent)	~200 nm	10-15	0.15	Not discussed	16	Liquid	18
Sonication	~few microns	1-5	1 (7~12% by recycling sediment)	~28	-		11
Ball milling with stearic acid surfactant of cetyltrimethyl ammonium bromide	1.7-3 µm	multilayer	10-29	Not discussed	-		19
Shear exfoliation	300-800 nm	5-8	3% achieved by recycling sediment	~9	84.6		14
Urea-assisted aqueous exfoliation	100-700 nm	~4	2.4	~12	100		20
low-power sonication for long times	1 µm	2.89	4	~21	346		21
Micro fluidization	1.9 µm	8.5 nm	100	Not discussed	223.5		22

Ball milling with PEI water solution	~450 nm	2-8	Not discussed	~4	~90		23
Wet-Jet Milling	~460 nm	3.2	~100	~15	143.8		24
Ball milling with Amino Acids water solution	1.46 µm	1−7	94.50	Not discussed	365		25
Acoustofluidic Exfoliation	100 nm	~7-9	2	~2.5	~12.5		26
Ball milling with melamine	~500 nm	3-4 layer	30	Not discussed	142.9	Solid	27
Ball milling with triazine/benzene derivative	Not discussed	Not discussed	5-33	Not discussed	-		28
Ball milling with solid diluents	200-400 nm	8	Not discussed	Not discussed	37.5		29
Ball milling with Amphotericin B	1 µm	10 nm	Not discussed	Not discussed	100		30
Salt-assisted ball milling	277-366 nm	1	10	100	321.5		31
Ball milling with glucose	120 nm	2-3	47	Not discussed	48		32
Ball milling with sugar	549 nm	4.4 nm	77	~10	124.8		33
Ball milling with stearic acid	1 µm	1-4 nm	85–90	Not discussed	400		34
ball milling with dry ice	100-500 nm	<5	Not discussed	~25	120	gas	35
Ball milling with SO_3	200-700 nm	1-3	Not discussed	19	200		36
Hydrothermal Exfoliation	2−4 µm	7−8	Not discussed	~6	400	others	37
Electrochemical Exfoliation	1-2 µm	2.1 nm	Not discussed	Not discussed	714.3		38
Flash joule heating	0.5-1.2 µm	1	80-90	Not discussed	850		39
Sticky exfoliation 15 h	~190 nm	0.8-0.9 nm	90.7 (Actual yield: ~78.3)	97.9%	190	Viscous PEI	This work
Sticky exfoliation 10 h	~780 nm	0.7-0.8 nm	86.3 (Actual yield: ~74.8)	91.2%	780		
Sticky exfoliation 5 h	~1640 nm	0.5-0.6 nm	52.4 (Actual yield: ~46.1)	76%	-		

Supplementary Section 3. Practicability

Supplementary 3.1 Sale-up study

Supplementary Table 3.1. The apparent yield of exfoliation with different graphite and PEI addition. All experiments were conducted at 500 rpm for 15h.

Graphite (g)	PEI (g)	Apparent yield (%)
5	5	37.8
	7.5	70.6
	10	42.3
10	7.5	54.7
	10	65.2
	12.5	15.6
20	10	51.4
	15	39.3
	20	8.6

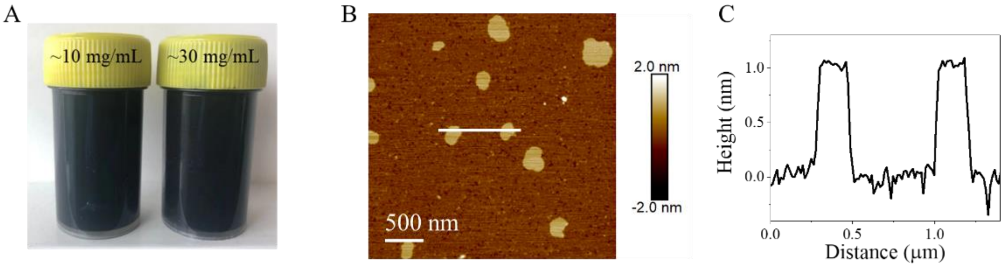

Supplementary Fig. 3.1. Scale-up study. (A) Photograph of the obtained graphene nanosheets being dispersed in water at different concentration. (B) Selected AFM image and (B) the height profile of the graphene obtained from scale-up experiments (Graphite 20g, PEI 10g, milling speed 500 rpm, milling time 15h).

The scale described in the main text used four 250 mL milling jars, and 0.5 g graphite was mixed with 2 g PEI in each jar. The ultra-high yield of the method and excellent quality of the resultant graphene were demonstrated as above. Sale-up studies were conducted by using 500 mL milling jars. We found that with more graphite added to the milling jars, less PEI was needed to ensure a decent yield (Supplementary Table 3.1). The PEI:graphite ratio could be lowered to 1:2 when 20 g graphite was added. The obtained nanosheets at the scale of 20-g graphite were analyzed in terms of size and thickness by AFM. Their lateral sizes (around 200 to 500 nm) are quite close to those of graphene nanosheets fabricated at small scale. The thickness of the graphene nanosheets indicated by AFM height profile shows 90% of them at around 1 nm, meaning they are monolayer as well (Supplementary Fig. 3.1).

Since there are four jars in this mill, 80 g graphite can be processed in one batch. Hence, 41g monolayer graphene was produced using the lab-scale ball milling machine. Considering that 15 h is taken for each batch, it gives a production capacity of ~2.74 g/h (~65.8 g/day). This means that only 20 lab-scale ball milling machines are needed to achieve a production capacity of kilogram-scale, which is needed for various industrial applications of graphene[40]. Given ball milling is a commonly used technology in industry with various scales, it is reasonable to believe that the sticky milling method has a great potential for large-scale production of high-quality monolayer graphene nanosheets. It should be noted that it is not feasible for mass production by simply multiplying the loading amount of graphite and PEI. The ratio of graphite to PEI is a key factor that should be carefully optimized when scaling up this method on practical occasions.

Supplementary 3.2 Dispersibility of graphene nanosheets in different solvents

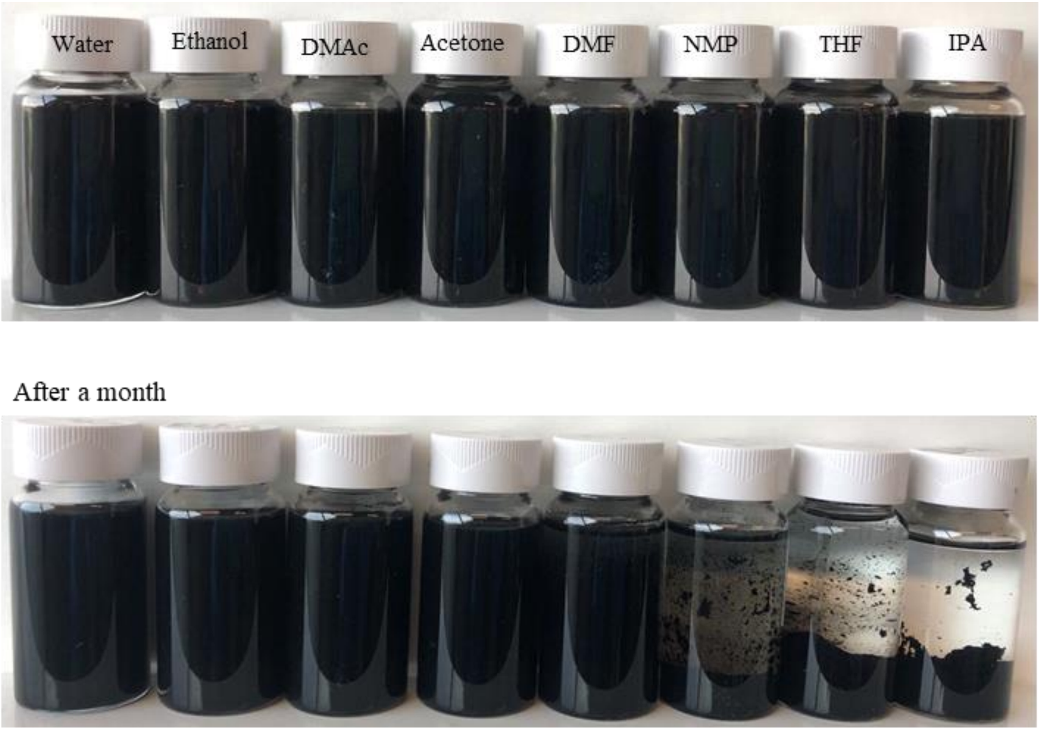

Supplementary Fig. 3.2. Dispersibility of graphene nanosheets. Photographs of graphene nanosheet dispersions in different solvents with a concentration of 0.5 mg/mL and after being stored for a month.

Apart from water, the as-prepared graphene can be well dispersed in many other commonly used solvents including Ethanol, Dimethylacetamide (DMAc), Acetone, N-Methyl-2-pyrrolidone (NMP), Tetrahydrofuran (THF), Isopropanol (IPA), and Dimethylformamide (DMF). These solvent dispersions were prepared by diluting the obtained water dispersion (~30 mg/mL) directly using specific solvents after conducting required rinsing process. As shown in Supplementary Figure 3.2, graphene can be well dispersed in these common solvents. Four suspensions of them (water, ethanol, acetone, DMAc) can be stored for a month without significant precipitation. The un-settled graphene

nanosheets in the solutions were monitored after a storage period of fortnight and a month, respectively (Fig. 2a in the main text). To measure the concentration of un-settled graphene nanosheets, 10 mL of solution was carefully pipetted out from the dispersion, and then filtrated on a pre-weighed nylon membrane (pore size: 0.22 µm). The membrane with nanosheets was dried in a 60 °C oven overnight and weighted to calculate the concentration of un-settled graphene. For the case of DMAc, which may remain in the membrane because of its high boiling point, we diluted it with 200 mL mill-Q water before filtration and washed it with another 200 mL water after filtration.

Supplementary 3.3 Drying for storage and Re-dispersion

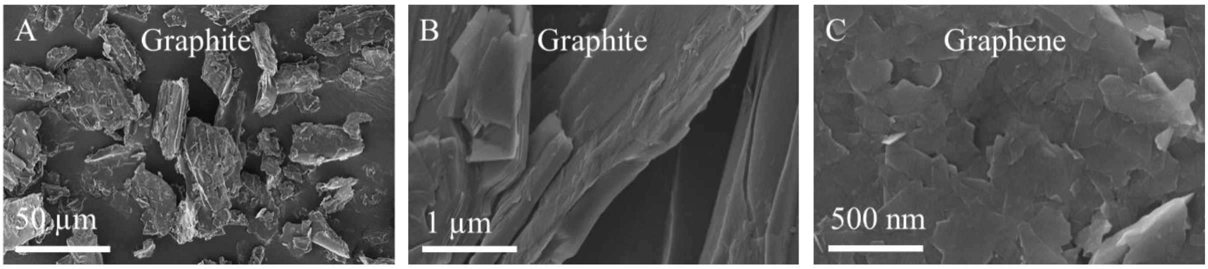

Supplementary Fig. 3.3. SEM images of the graphite raw materials (A, B) and graphene powder (C)

For convenient storage and distribution, it is highly desired for graphene products to be processed into powder form. Graphene prepared by liquid phase exfoliation usually relies on organic solvents as a suspension medium, which causes inconvenience in practical storage, transport and distribution[41]. To combat this, it is meaningful to explore whether the graphene nanosheets synthesized in this study can be processed into powder for storage and distribution, and then re-dispersed for late use. To achieve this, powder of graphene was first obtained by freeze-drying graphene water dispersion with a concentration of 2 mg/ml. The powder was then kept in a plastic or glass sample container in an ambient environment. The powder shows an extremely fluffy texture with a noticeable increase in volume compared to the graphite raw materials (Fig. 1a in the main text). SEM images show that graphite displays as many separated particles as possible with their size at tens of microns (Supplementary Figs. 3.3 A-B). In contrast, the dried graphene powder shows a clearly identifiable 2-dimensional structure (Supplementary Fig. 3.3 C).

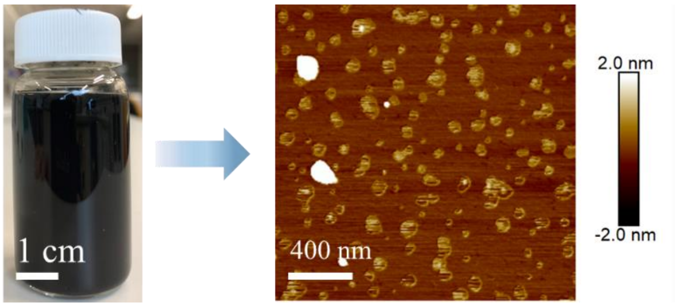

Supplementary Fig. 3.4. Photos of re-dispersed graphene in water/ethanol (1:1, v/v) mixed solvent and their size and thickness distribution observed by AFM.

Although graphene in powder is ideal for storage and transport, liquid form is widely needed in material processing, e.g., mixing, filtration, coating and inkjet printing, and so on. Hence, a more important property of the synthesized graphene nanosheets is whether they can be re-dispersed in a liquid medium after they are dried forming powder. After the graphene powder (exfoliated with PEI Vis-5, milling time 15h) was kept under the ambient environment for three months, we tried to re-disperse them using different solvents. Briefly, 2-mg graphene was mixed with 20-mL dispersant, and the mixture was then subject to sonication for 30 min. The top 10 mL dispersion was then pipetted out and filtrated onto a pre-weighed nylon membrane to measure graphene concentration. The experiment results showed that around 36% of graphene powder can be re-dispersed in pure water after 30 min sonication forming a graphene solution of 0.36 mg/mL. Surprisingly, we found that the dried graphene powder could be easily re-dispersed after 30 min of sonication into a mixed solvent of water and ethanol (1:1), forming a graphene solution of 0.83 mg/mL, shown in Supplementary Figure 3.4. AFM imaging was applied to confirm the quality of graphene nanosheets after dry storage and re-dispersion. As shown in Figure 3.4, the re-dispersed graphene nanosheets show a lateral size at around 200 nm and apparent height at around 0.8 nm, remaining their high quality after freeze-drying and re-dispersion processes.

Supplementary Section 4. Residual PEI

Supplementary 4.1 Concentration of residual PEI

Graphene powder samples were prepared by freeze-drying the water dispersions after water rinsing. An elemental analyzer (FlashSmart, Thermo Scientific) was adopted to analyze the weight ratio of elemental content (C, N, and H) in these graphene powder samples synthesized at different exfoliation time. In this work, we mainly focused on the weight ratio of N, which is only contributed from PEI. The N content of PEI Vis-5 is of 31.95 wt.%. Therefore, the weight ratio of residual PEI on graphene can be obtained through simple calculation. As shown in Supplementary Table 4.1 and Supplementary Figure 4.1, the weight ratio of residual PEI increases along with milling time, but slowly levels off. We speculate that PEI molecules were grafted or bonded onto graphene nanosheets largely due to the fragmentation and breaking of graphite. When graphite breaks caused by the crashing or shearing effects of grinding balls, numerous active C atoms are created along newly formed edges due to the breaking of C-C bonds. These active C atoms can react with amino groups of PEI molecules driven by high mechanical energy, forming strong covalent bonds[42].

Supplementary Table 4.1. The weight ratio of residual PEI on the graphene nanosheets exfoliated at different milling time by PEI Vis-5. All the number denoted the mass percentage (%)

Milling time (h)	Nitrogen element (wt.%)	Deviation	Grafted PEI (wt.%)
1	1.39	0.1	4.35
5	2	0.3	6.26
10	3.67	0.2	11.49
15	3.99	0.2	12.43
PEI Vis-5	31.95	0.6	-

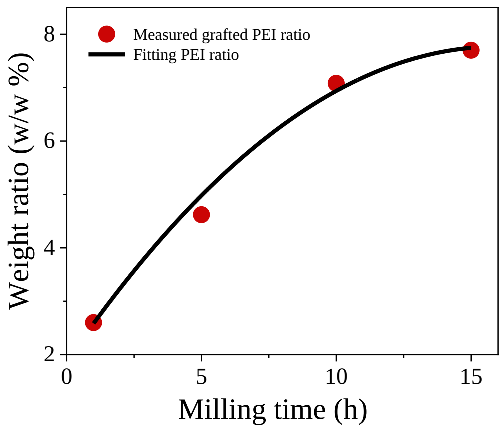

Supplementary Fig. 4.1. Trend graph of the amount of residual PEI on graphene nanosheets as the function of exfoliation time (based on Supplementary Table 4.1). Symbols are experimental data; line is obtained by a nonlinear fitting.

Supplementary 4.2 Removal of residual PEI

Although residual PEI on graphene nanosheets offers great convenience in storage and redispersion, they may be not desirable on some occasions. Therefore, two methods were explored in this study to remove residual PEI molecules. PEI is a polycation and its ionization strongly depends on pH[43]. Normally, the higher the ionization degree, the better the solubility in water. In light of this, the first method is acid treatment. In this method, graphene (exfoliated with Vis-5 for 10h) dispersion was diluted in 0.1M HCl solution to a concentration of 0.1 mg/mL. The solution then was put on a shaker overnight and followed by thoroughly washing with Milli-Q water. After being re-dispersed in water and freeze-dried, the as-obtained graphene powder was characterized by an Elementar Vario EL III elemental analyzer in terms of its elemental composition, in particular, the element weight ratio of N. Details are provided in Supplementary Section 4.1. The results show that residual PEI was partially (~45%) removed by acid treatment (Supplementary Table 4.2).

According to the results of TGA, PEI rapidly decomposes between 200 to 420 °C under the flow of inert gas. In this context, the second method is thermal treatment. Graphene powder was calcined in a tube furnace under nitrogen flow. The experiment was conducted by heating the graphene sample to 600 °C with an increment of 5 °C·min^{-1}. It was then stayed at 600 °C for 30 min before lowering to room temperature. The calcined powder was then analyzed by an elemental analyzer following the same method described in section 4.1. The results show around 98% of residual PEI molecules on graphene nanosheets were removed by thermal treatment (Supplementary Table 4.2).

Supplementary Table 4.2. The weight ratio changes of residual PEI after acid and thermal treatment.

Sample	Elemental analysis		XPS	
	Nitrogen (weight ratio%)	Grafted PEI (wt.%)	Nitrogen (atomic %)	Grafted PEI (wt.%)
PEI Vis-5	31.95	-	30.75	-
Exfoliated graphene	3.67	11.49	3.53	12.07
HCl-treated graphene	2.01	6.29	2.57	8.79
Thermal-treated graphene	0.08	0.25	0.57	1.85

Apart from elemental analysis, the removal of PEI functionalities was also investigated by X-ray photoelectron spectroscopy (XPS). Full XPS survey scans of pristine graphene powder, de-functionalized graphene power, and pure PEI are provided as Supplementary Figure 4.2. The corresponding nitrogen atomic percentages are calculated by Thermo Scientific Advantage software using the auto peak fitting function and a standard Shirley background was used for all spectral regions. The nitrogen concentration calculated from XPS is in line with the result of elemental analysis (CHNS) for samples with high nitrogen content such as PEI and pristine exfoliated graphene (Supplementary Table 4.2). However, the difference between the results of the XPS and CHNS analysis rises with the nitrogen contents in the samples approach the detection limit, especially for the thermal-treated graphene. The difference between the results of the two methods may stems from the difference of their sample preparation methods and analyzing mechanisms. For the CHNS analysis, bulk powder was completely oxidized by “flash combustions”. The combustion products are separated and analyzed to give quantitative information on components in the samples[44]. By comparison, graphene was deposited on a substrate and shot by the X-rays for the XPS analysis. The qualitative and quantitative signals are only generated and analyzed from the sample surface[45, 46]. In summary, the PEI removal efficiency of acid washing is at 45.3%, and 27.2% measured by the CHNS and XPS analysis, respectively, which can be further increased to 97.8% (CHNS) / 84.7% (XPS) by applying thermal treatment.

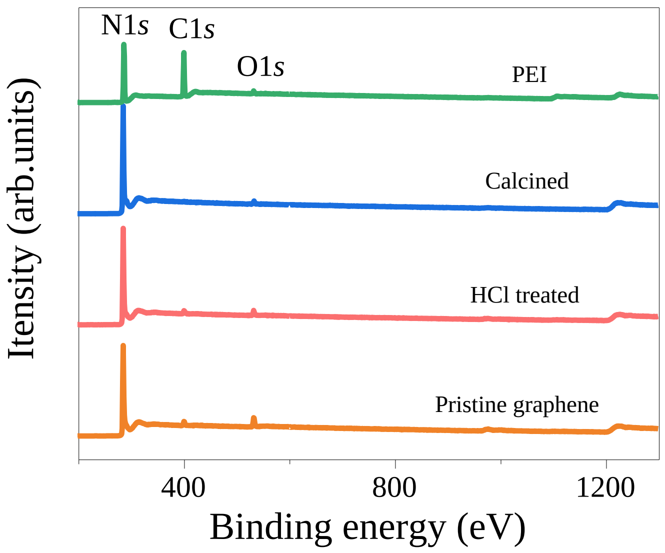

Supplementary Fig. 4.2. X-ray photoelectron spectroscopy (XPS) full survey scans of PEI, pristine graphene, HCl-treated graphene, and calcined graphene.

Supplementary 4.3 Conductivity of graphene nanosheets

Supplementary Table 4.3. Sheet-resistance and the calculated conductivity of the graphene films.

Sample	Thickness (T)	Free-standing	De-functionalization	Sheet-resistance (Ω/sq^{-1}, Q)	Conductivity (S/m, 1/QT)
1	1.6 μm	No	No	43.29	14,438
2	9.1 μm	Yes	No	6.84	16,066
3	18.2 μm	Yes	No	1.96	28,033
4	15.4 μm	Yes	HCl treated	1.37	47,398
5	12.6 μm	Yes	Calcined	0. 246	322,000

Graphene obtained by 10-h milling with PEI Vis-5 was chosen for fabricating conductive films. Graphene water dispersion was diluted to a concentration of 0.01 mg/mL and then was vacuum-assisted filtrated on PES substrate membranes. The thickness was controlled by varying the loading density. A source meter equipped with a four-point probe was applied to measure the sheet resistance of the obtained films. For thin membrane like sample 1, the conductivity was measured with the PES substrate. For thicker films, free-standing graphene films were obtained by following a freeze-and-sublimation transfer strategy[6]. Noted that graphene loading density of samples 3, 4, 5 is at the same level but with different post-treatments. The obtained films showed different thicknesses. The PEI functionalities were partially and totally removed to study its impacts on film conductivity.

Procedures for removing PEI and the preparation of samples 4, 5 are detailly described in Supplementary Section 1.8. The results show the conductivity of the obtained graphene is reasonably high, with the conductivity of thick film can reach 28,033 S/m. The conductivity can be further boosted to 47,398 S/m and 322,000 S/m, respectively, by acid or thermal treatment to partially or completely removal of residual PEI functionalities. This high conductivity of the as-prepared graphene show clear superiority over its reported counterparts including GO (4,000-7,000 S/m)[47, 48, 49] and sonication-induced graphene (1,200-6,500 S/m)[11, 50, 51] films.

Supplementary 4.4 Zeta potential of synthesized nanosheets

Zeta potential of the exfoliated nanosheets (graphene, BN and g-C_3N_4) was tested from their diluted water dispersion at a concentration of 0.05 mg/mL. Free PEI molecules were washed out before testing. pH of these solutions was also measured. All Zeta potential tests were performed directly using the water dispersion without pH adjusting. Because of residual PEI, the exfoliated graphene, h-BN and g-C_3N_4 all possess positive zeta potentials with ultrahigh values of over 50 mV. This positive-charged property stands as a clear contrast to the nanosheet dispersions obtained from other exfoliation methods (Supplementary Table 4.4.).

Supplementary Table 4.4. Comparison on Zeta potential of the nanosheet water dispersions in this work with reported nanosheet water dispersions.

materials of nanosheets	Exfoliation Method	Zeta potential (mV)	pH	Reference
BN	Oxidation + Sonication	-21.4	-	52
	Ball milling with sugar	-36.7 to -45.7	~9	53
	Ball milling with 2-furoci acid	-45 to 47.5	8 to 9	54
	Ball milling with urea	~34	-	55
g-C_3N_4	Mechanical grinding	-35.4		56
	Ball milling	-28.2	-	57
	Sonication in water	-27	7	1
Graphene	Shear with NaOH	-30	7 to 9	58
	Sonication with Urea	-38 to -41	-	20
	Ball milling with salts	-30	7	31
	Ball milling with reactive gases	-32.8 to -48.6	-	42

	Ball milling with dry ice	-34.2	-	[35]
	Hydrothermal Exfoliation	-38.26	-	[37]
	Ball milling with amino acid	-56.3	10	[25]
	Ball milling with SO_3	-32 to -58	-	[36]
PEI Vis-5		22.4 ± 1.2	10.2<br>3	
graphene	Sticky exfoliation by PEI Vis-5 for 15 h	52.9 ± 2.4	6.12	This work
Exfoliated h-BN		52.7 ± 2.6	5.99	
Exfoliated $g\text{-}C_3N_4$		53.8 ± 1.7	7.05	

Supplementary 4.5 Ion transport of HCl-treated membranes

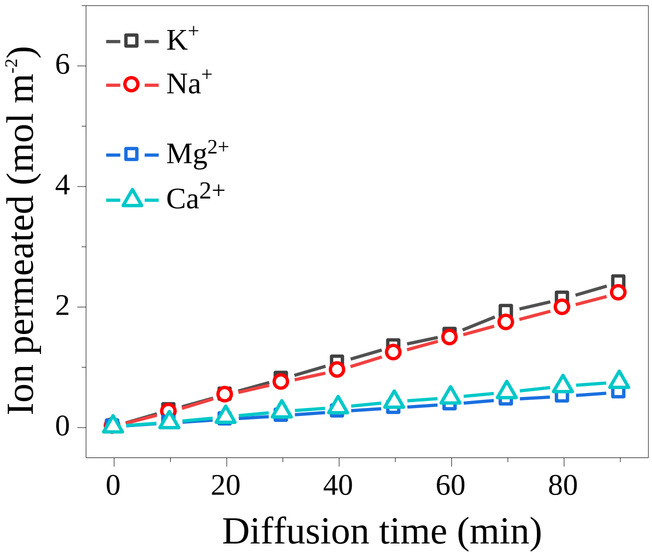

Supplementary Fig. 4.3. Ion permeation rate of HCl-treated membranes

We found that HCl-treated membranes exhibited a diffusion rate of monovalent ions (Na^+, K^+) at ~1.9 mol m^{-2} h^{-1} and divalent ions (Ca^{2+}, Mg^{2+}) of ~0.5 mol m^{-2} h^{-1}, leading to a moderated mono/divalent ion selectivity of ~2.9 to 4.4 (Supplementary Fig. 4.3). The decreased ion transport rate and divalent/monovalent ion selectivity emphasize the main roles of PEI in the laminar membrane[4, 59, 60]: (1) acting as molecular spacers to increase the mass transport rate; (2) regulating ion transport by the Donnan effect.

Supplementary Section 5. Exfoliation mechanism

Supplementary 5.1 Graphene at intermediate stage

To understand the exfoliation mechanism, graphene obtained from 1-h and 2-h milling was deposited on mica by following the same AFM sample preparation procedures excepting without centrifugation. We found that both thin-yet-large and thick-yet-small nanosheets exist in the exfoliation products at 1-1 and 2-h (Supplementary Fig. 5.1). The absence of large and thick particles could be the result that they are prone to precipitation and thus being excluded during sample preparation process. Along with the milling time increasing to 2 h, the lateral size of the nanosheets narrows down to 1 to 2 µm, and their thickness reduces to less than 100 nm (Supplementary Fig. 5.1).

According to these results, the proposed exfoliation and material breaking mechanism presents as follows. Starting from thick and large particles, the in-plane breaking and out-of-plane delamination occur simultaneously on them at the initial stage driven by grinding effects. The delamination process could start from the very top/bottom surface of these bulk particles, delivering ultra-thin nanosheets and leaving thick parent particles. With further grinding, chances of breaking will dramatically be reduced with the deceasing thickness or when the lateral size of the nanosheets is comparable to the surface roughness of the grinding balls (Supplementary Fig. 5.6). As a result, the lateral size distribution narrows with the prolonging of milling time. The exfoliation is further evidenced by an incompletely exfoliated graphene shows height steps of ~0.7 nm (Supplementary Fig. 5.2)

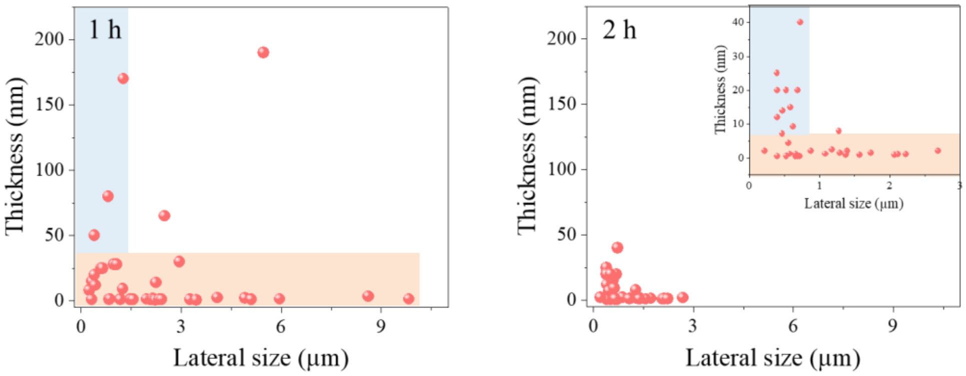

Supplementary Fig. 5.1. **Statistical analysis on the graphene products in terms of the lateral size and thickness obtained by 1-h milling and 2-h milling based on AFM characterization.**

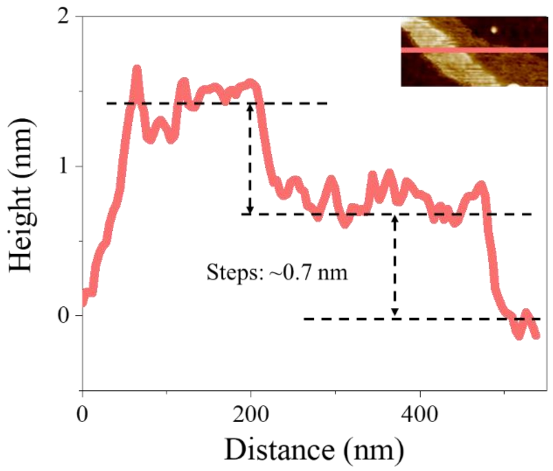

Supplementary Fig. 5.2. AFM step heights of a graphene nanosheet spotted from the 2-h milling product. The bilayer graphene shows a step height of ~0.7 nm, corresponding to the measure thickness of monolayer graphene.

Supplementary 5.2 DEM simulation

Supplementary 5.2.1 Governing equations

The discrete particles are described by the discrete element method (DEM)[61, 62, 63]. A particle has two types of motion: translational and rotational. While moving, the particle may interact with its neighboring particles and/or wall boundary, through which the momentum and energy exchange takes place. At any time *t*, the equations governing the motion of particle *i* of mass m_i and radius R_i can be written as:

$$m_i\, dv_i/dt = \sum_j (f_{e,ij} + f_{d,ij} + f_{vis,ij} + f_{c,ij}) + m_i g, \tag{5.1}$$

and

$$I_i\, d\omega_i/dt = \sum_j (T_{t,ij} + T_{r,ij}), \tag{5.2}$$

where $\mathbf{v}_i$ and $\boldsymbol{\omega}_i$ are the translational and rotational velocities of particle *i*, and I_i ($= 2m_iR_i^2/5$) is the moment of the inertia of the particle. The forces involved are: the gravitational force $m_i\mathbf{g}$ and the forces between particles (and between particles and walls) which include the elastic force $\mathbf{f}_{e,ij}$, damping force $\mathbf{f}_{d,ij}$, viscous force $\mathbf{f}_{vis,ij}$ and the cohesive force $\mathbf{f}_{c,ij}$. The torque acting on particle *i* due to particle *j* includes two components: $\mathbf{T}_{t,ij}$ generated by the tangential force and $\mathbf{T}_{r,ij}$ generated by

asymmetric normal contact force. If particle *i* undergoes multiple interactions, the individual interaction forces and torques are summed up for all particles interacting with particle *i*. In this work, the cohesive force is the capillary force due to the liquid PEI. The model for the capillary force considering the normal and tangential viscous forces is adopted here[64]. Most of the equations have been well established as, for example, reviewed by Zhu et al[65].

To accommodate the complicated geometry boundary and the rotational motion of the mill, an in-house DEM package is used[62]. Meshes are generated for the mill, and the contacts between the mill and particles can be detected and treated similarly to particle-particle contacts. The DEM package has been applied to different granular systems and proved to work[62, 63, 66].

Supplementary 5.2.2 Parameters for the DEM simulation

The geometry of the mill is set according to the 250 mL milling jar where the diameter is 80 mm and the height 78 mm. The revolution speed is set as 500 rpm. Three sizes of the grinding balls (10 mm, 5 mm, and 1 mm in diameter) are considered and their numbers are 29, 450, and 3,000. The density of grinding balls and the mill is 3.9 g/cm^3 and the passion ratio is 0.23. PEI properties are set according to the experimental measurement. The force analysis was conducted after the simulation reaches a steady state after ~ 20 seconds.

Supplementary 5.2.3 Results and discussion of DEM simulation

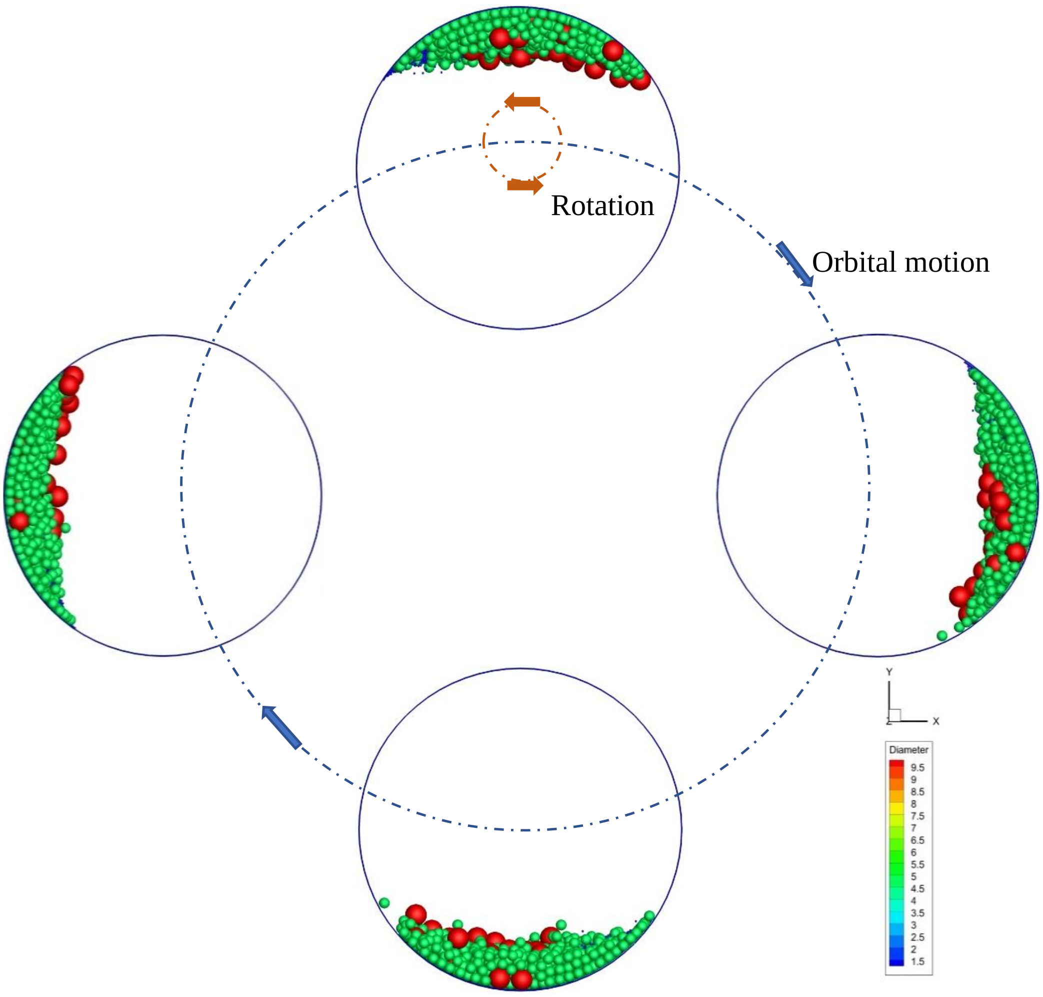

Supplementary Fig. 5.3. The simulated positions of grinding balls during a full rotation

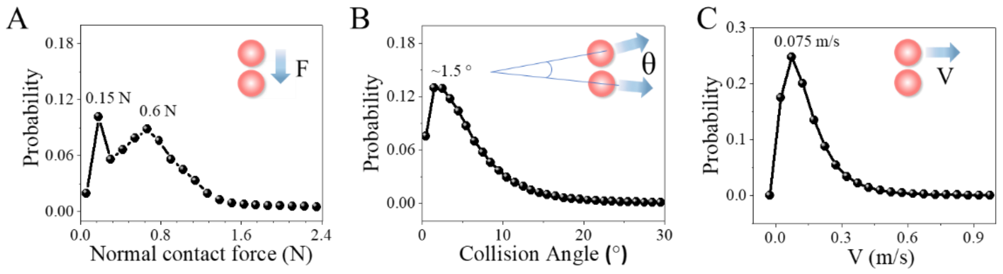

Supplementary Fig. 5.4. DEM statistical analysis. (A) Statistic analysis of the normal compression force, (B) angle of relative motion, and (C) relative velocity of motion between grinding balls during operation by Discrete Element Method (DEM) simulation study.

The grinding balls in the planetary mill move collectively as shown in the Supplementary Figure 5.4, which is different to a normal rotating ball mill. In the planetary mill, there is no drastic collision as in an ordinary ball mill, in which the balls are moving together under the strong centrifugal force related to the orbital revolution of the mill. However, when one checks the position of balls in the planetary mill it will be found that there is relative and mild rolling and sliding motions between balls

due to the rotation of the mill, generating normal force (0-2 N) and relative velocity (0-0.6 m/S) (Supplementary Figs. 5.3 A, C). Statistical analysis shows grinding balls almost move parallelly with the angle between their moving directions in the range of 0-10° (Supplementary Fig. 5.4B). leading to the grinding and delamination of the two-dimensional materials added to the mill.

To understand the intralayer breaking, the normal compression force between two colliding grinding balls and the surface roughness morphology of the grinding ball are analyzed. The distance between rough peaks (or tips) is about 3 microns (Supplementary Fig. 5.6), and the most probable normal compression force is about 0.6 N. For a cone shape of the rough tip, the normal compression force can generate a large enough stress (~600 GP) for the intralayer breakage, obtained with the cross-sectional area of the rough tips at the half height of the cone (the diameter of the area from the measurement is 0.83 to 1.16 micron).

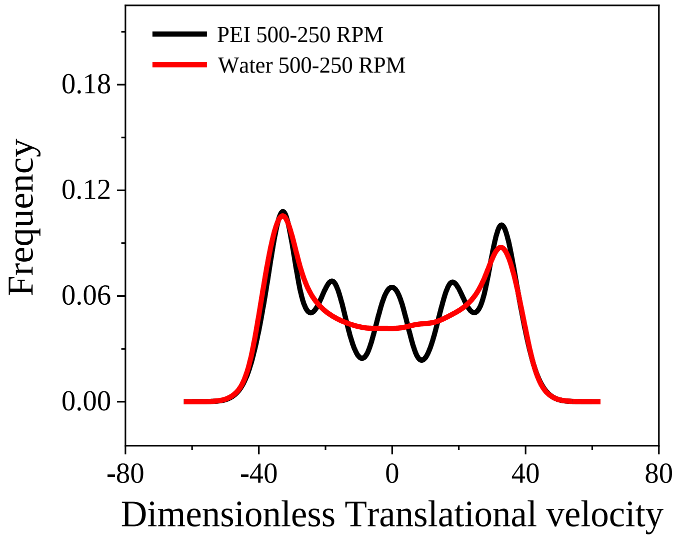

Supplementary Fig. 5.5. **The transitional velocity distribution of grinding balls during operation with addition of water and PEI respectively.**

To simplify the calculation, we kept the moving behavior of grinding balls constant when analyzing the system with and without PEI addition. In order to verify the reliability of this assumption, we did a comparison of the transitional velocity distribution of grinding balls with adding water and PEI respectively. It is noted that the systems with water and PEI have a similar distribution. The system

with PEI has obvious fluctuations between the two peaks which could be the result of increased viscous energy dissipation. This difference is expected to have little effect on the exfoliation.

Supplementary 5.3 Surface morphology characterization of grinding balls

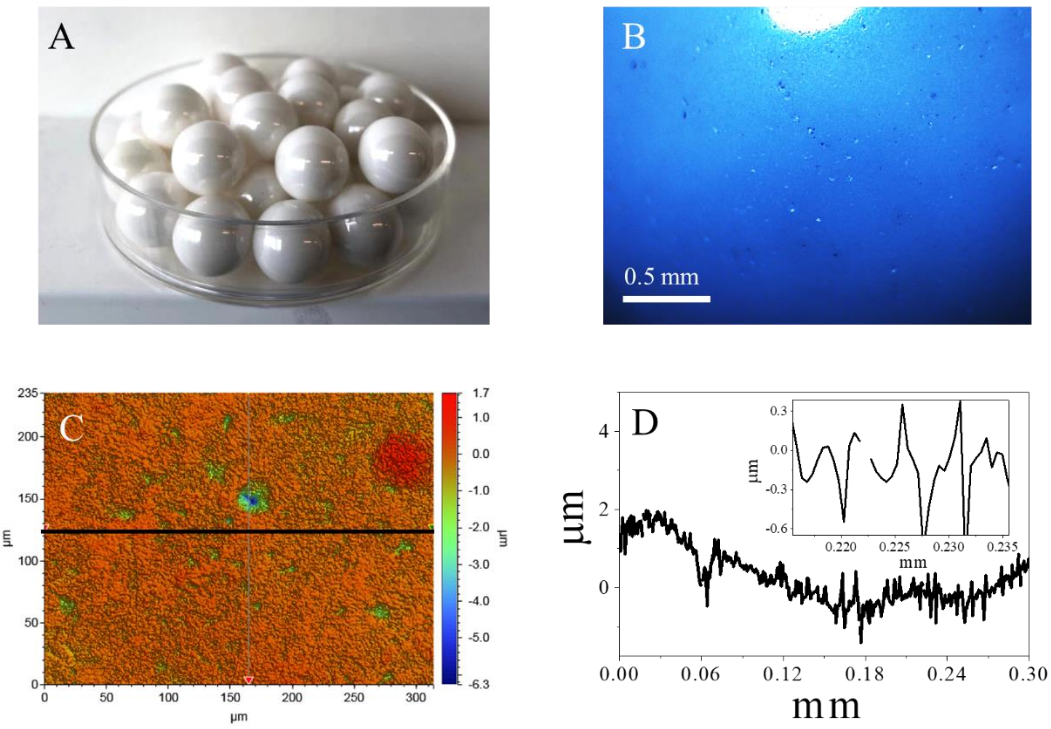

Supplementary Fig. 5.6. **Analysis on milling balls.** (A) Photograph of the ZrO_2 grinding balls (d=10 mm). (B) Surface morphology of the grinding balls observed by optical microscope. (C) Surface roughness of the grinding balls (the smallest one, d=1 mm) characterized by an optical profilometer. (D) Height profile along the black line in C.

Although the balls look glossy on the surface, there are actually highly rough on the surface. There are many micron-scale holes on the surface that can be seen by optical microscope (Supplementary Fig. 5.6B). An optical profilometer (Contour GTI 3D optical profiler, Bruker) was adopted to give more details of the milling-ball surface. The largest grinding balls (d=10 mm) were chosen for the characterization. The results show that the surface presents as a ridge-and-valley structure. Statistical analysis of 50 peaks on the height profile along the black line in Supplementary Figure 5.5C shows that the half peak width of these tips is average at 1.6 µm, the height of these tips is averaged at 0.388 µm, and the distance between neighboring tips is averaged at 2.92 µm.

Supplementary 5.4 Effect of PEI:graphite ratio

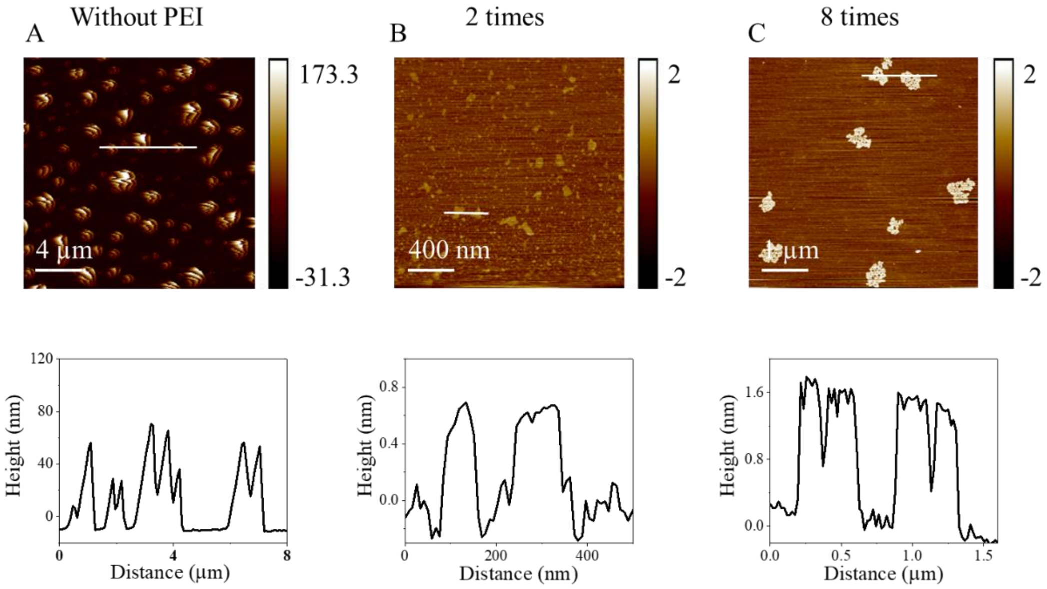

Supplementary Fig. 5.7. **AFM images and the height profile along the white lines of exfoliated graphite without PEI** (A), PEI:graphite ratio = 2 (B), PEI:graphite ratio = 8 (C).

To evaluate the influence of PEI:graphite ratio, the amount of PEI was adjusted with other parameters keeping consistent (PEI Vis-5, milling time 15h). Only amorphous carbon nanoparticles without distinct 2D characteristics were observed when milling graphite alone (Supplementary Fig. 5.7A). When the PEI:graphite ratio was set as 2, the obtained nanosheets exhibited a thickness of ~0.6 nm (Supplementary Fig. 5.7B), indicating they are monolayer graphene. However, their lateral size shows a broad distribution from tens of nanometers to hundreds of nanometers. This is the result that inefficient protection of PEI to graphene nanosheets from inadequate PEI addition. When we increased PEI addition to 8 times of graphite, the lateral size of graphene increased by 2 to 4 times to ~400-800 nm. However, their thickness only reduced to 1.5 to 1.7 nm (Supplementary Fig. 5.7C), meaning inefficient exfoliation when adding excessive PEI. This phenomenon can be well described by our two-plane model. Excessive PEI buffer layer can provide effective protection to nanosheets, which enables graphene with a decent lateral size. However, according to equation (1) in the main text, shear force F is inversely proportional to the thickness of buffer layer Y, which means the thicker the buffer layer, the smaller shear force applied on graphite for exfoliation. As a consequence, 8 times addition of PEI is only able to exfoliate the graphene to ~1.5 nm in a period of 15 h. The results indicate that there is an optional PEI:graphite ratio.

Supplementary 5.5 Density Functional Theory (DFT) calculation

Supplementary 5.5.1 Calculation methods

We performed density functional theory (DFT) calculation using the Vienna ab initio simulation package (VASP)[67, 68]. The Perdew, Burke, and Ernzerhof (PBE)[69] exchange-correlation functional

and the projector augmented wave (PAW) approach were adopted[70]. The DFT-D3 semi-empirical method is used to describe the weak dispersive force[71]. In our DFT calculations, the plane wave basis set cutoff energy was 400 eV. The energy convergence criterion was set to be 10^{-4} eV, and the residual forces in the converged structures are smaller than 0.01 eV/Å. The valence atomic configurations are $4p^6 5s^1 4d^5$ for Mo and $5p^6 6s^2 5d^4$ for W. A vacuum space in z direction was larger than 10 Å to minimize the spurious interactions between the periodically repeated images. The model size of the monolayer supercell of 2D materials was greater than 30 Å × 30 Å and a Γ-centered 1×1×1 k-point mesh was used for the PEI/PEG adsorption on 2D materials monolayer. The k-point grid was 3 × 3 × 1 for the bulk and bilayer of the 2D materials. At a chosen relative top layer displacement, the x and y coordinates of all atoms were fixed and only the z coordinate was relaxed to generate the potential energy surfaces. In the cases of C_3N_4 and COF, the bilayers were set as rigid layers to avoid structural deformation during the sliding motion

Supplementary 5.5.2 Results and discussion of DFT

Density functional theory (DFT) calculations were performed to explore the effect of PEI in 2D materials exfoliation. To simplify the calculation, the number of repeating units(n) in PEI and PEG was set as 1 and 12 respectively, and thus the number of CH_2 unit(N) in PEI and PEG were 24 in our calculations

Firstly, we compared the interlayer binding energies (E_{binding}) of 2D materials with the adsorption energies (E_{ads}) of PEI/PEG on their monolayer. Graphene, COF, g-C_3N_4 and h-BN are considered in our calculation.

The E_{binding} is calculated by

$$E_{\text{binding}} = \frac{E_{bulk} - A \times E_{\text{monolayer}}}{A \times S} \quad (5.3)$$

where $E_{monolayer}$ and E_{bulk} are the total energies of 2D monolayer and bulk, respectively, A is the number of layers in bulk, and S is the area of the layer in the bulk. As shown in Supplementary Table 5.1, the E_{binding} value of COF is close to zero (-0.07 meV/Å^2), while the E_{binding} values of other 2D materials are between -15 ~ -35 meV/Å^2.

The E_{ads} per CH_2 unit is calculated by

$$E_{\text{ads}} = \frac{E_{\text{tot}} - E_{\text{monolayer}} - E_{\text{molecule}}}{N} \quad (5.4)$$

where E_{tot} and $E_{\text{monolayer}}$ are the total energies of 2D materials monolayer with and without adsorbed a single PEI molecule, respectively, E_{molecule} is the energy of a single PEI/PEG molecule in a vacuum, N is the number of CH_2 unit in PEI/PEG.

For a better comparability between E_{binding} and E_{ads}, we roughly estimate the adsorption energies per unit area $E_{\text{ads}}(PEI/PEG)$ by:

$$E_{\text{ads}}(\text{PEI}) = \frac{E_{\text{ads}}}{\pi\left(\frac{l_{\text{CH2-CH2}}}{2}\right)^2} \quad (5.5)$$

where $l_{CH2-CH2}$ is the distance between two neighboring CH_2 units. As shown in Supplementary Table 5.1, we found that the interaction of both PEI and PEG with studied materials is stronger than their interlayer binding strength. This result indicates that energetically both of PEI and PEG are suitable for exfoliation.

The potential energy surfaces of 2D materials show three-fold symmetry, and the bigger gradient means larger interface shearing force. Therefore, we can calculate the ISS value through the sliding direction which has the lowest gradient (Supplementary Fig. 5.8). The ISS (τ) is defined as

$$\tau = \frac{F}{S} = \frac{\frac{\partial E}{\partial x}}{S} \quad (5.6)$$

where F is the shearing force in the sliding process, S is the area of 2D materials bilayer, E is the total energy of 2D materials bilayer in the sliding process, and x is the sliding displacement of the top layer.

According to formula (5.5) and (5.6), we estimated the range of ISS value of these layered materials is 0.116 bilayer graphene (Supplementary Table 5.2), which is close to the previous reported 0.1 Gpa[72, 73]. The ISS values are smaller than the experimental shear force the sticky milling can achieve, so mechanical exfoliation is energetically possible.

Supplementary Table 5.1. The calculated adsorption energy of PEI and PEG-400 on the surface of studied materials.

Materials	Interlayer binding energy (meV/unit area)	PEI (adsorption, meV/unit area)	PEG-400 (adsorption, meV/unit area)
Graphene	-17.44985	-75.67521	-50.0092
TAPB-PDA COF	-0.07461	-117.64238	-37.95865
g-C_3N_4	-18.68304	-13880	-13840
h-BN	-20.39887	-67.23958	-56.94109

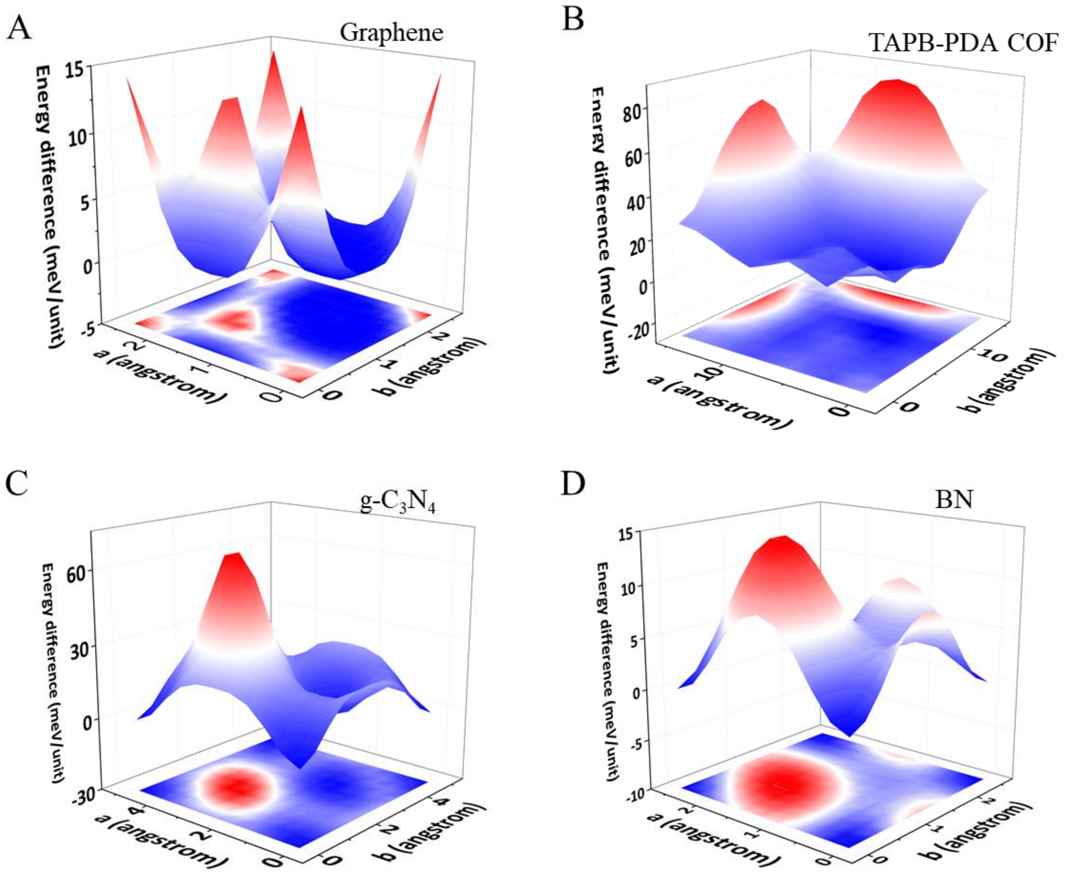

Supplementary Fig. 5.8. **Three-dimensional potential energy surfaces for the sliding motion of bilayer**. (A) graphene, (B) TAPB-PDA, (C) g-C_3N_4 (D) h-BN. The blue-white-red represents the energy difference relative the ground state.

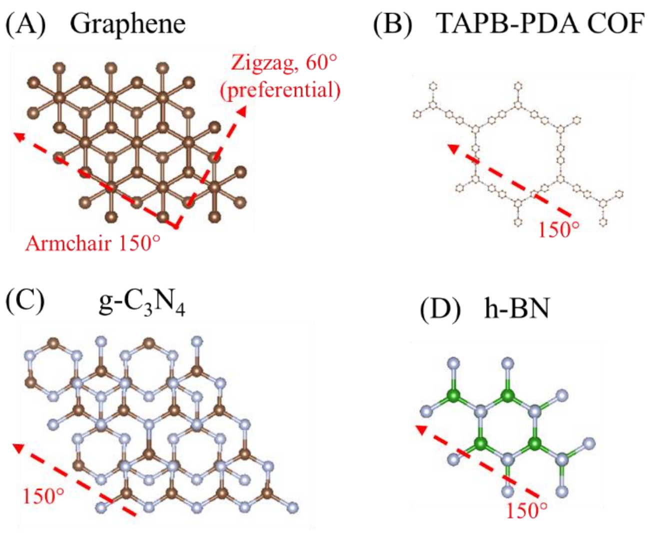

Supplementary Fig. 5.9. **The stacking order of the ground state of (A) graphene, (B) TAPB-PDA, (C) g-C_3N_4 (D) h-BN.** The red rows indicate the preferential sliding direction which has the lowest gradient on the potential energy surface. The most difficult direction (armchair) for graphene to slide is also provided for reference.

Supplementary Table 5.2. The range of maximum interface shearing force ($\frac{\partial E}{\partial x}$) and interface shearing strength (τ) of studied materials following the preferential sliding direction. Graphene sliding along the armchair direction with the highest shearing strength is also provided for comparison.

2D materials	$\frac{\partial E}{\partial x}$ (meV/Å)	τ (Maximum, GPa)
Graphene (zigzag)	3.82092	0.116
Graphene (armchair)	11.24192	0.342
TAPB-PDA COF	5.22257	0.001
g-C_3N_4	17.11973	0.139
h-BN	6.90088	0.190

Supplementary 5.6 Effect of viscosity of PEI mixtures

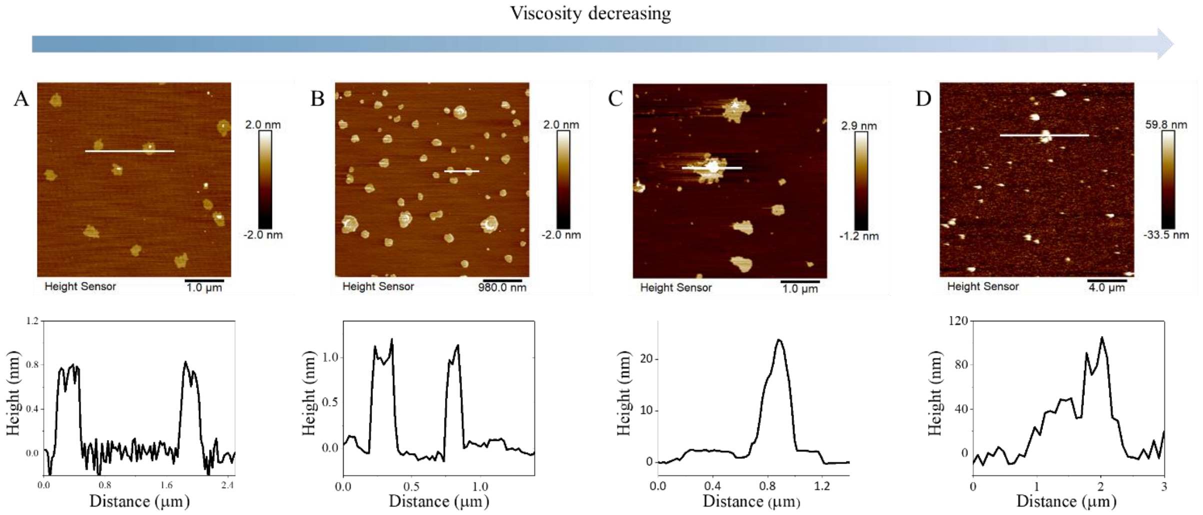

Supplementary Fig. 5.10. AFM images of the prepared graphene exfoliated using PEI mixture with different viscosity. (A) PEI Vis-4 (~33,716 mPa·s), (B) PEI Vis-3 (~16,858 mPa·s), (C) PEI Vis-2 (7, 235 mPa·s), (D) PEI Vis-1 (1, 508 mPa·s), respectively, at a milling time of 10 h. The right column is the corresponding height profiles along the lines in the images.

The products exfoliated using PEI Vis-5 (~150,882 mPa·s), Vis-4 (~33,716 mPa·s) and Vis-3 (~16,858 mPa·s) all have graphene nanosheets with apparent heights lower than 1 nm (Supplementary Figs. 5.10A-B). However, more and more multilayer graphene flakes with thickness ranging from 5 nm to tens of nanometers are observed when reducing the viscosity to 7,235 mPa·s (Supplementary Fig. 5.10C). The lateral size of these flakes exhibits a broad distribution from hundreds of nanometers to microns, meaning a significant crushing may occur resulting from insufficient protection. Further reducing viscosity to 1508 mPa·s, thick nanoplatelets with their thickness reaches hundreds of nanometers are detected and many of them are more like nanoparticles (Supplementary Fig. 5.10D). The results suggest a small amount of monolayer graphene can be obtained by PEI with a viscosity

of around 7235 mPa·s. However, a viscosity of over around 16,858 mPa.s is needed to achieve a total monolayer product. We therefore take 7235 mPa·s as the viscosity threshold, by which only the viscosity of the exfoliation liquid over this threshold can be seen as sticky exfoliation. The viscosity of the exfoliation liquid under this threshold is not suitable for the production of monolayer graphene, which could be the reason for the failure of achieving high monolayer percentage by traditional LPE methods.

Supplementary 5.7 Calculation of viscosity threshold for obtaining monolayer graphene

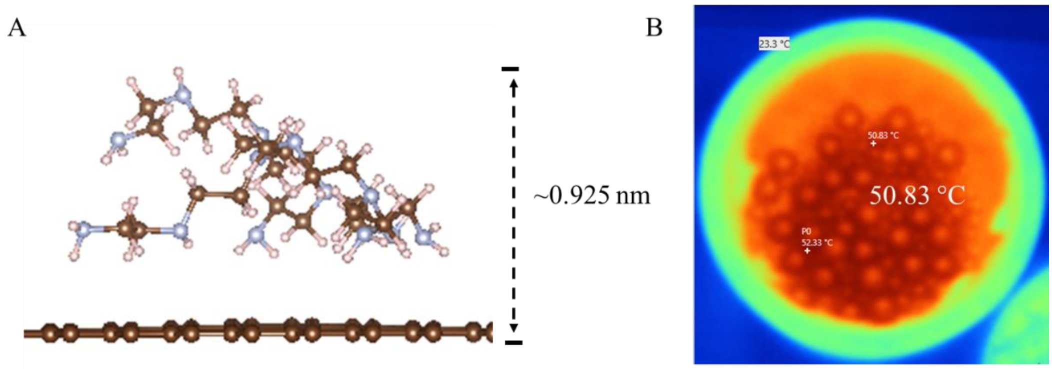

Supplementary Fig. 5.11. (A) Schematic of the thickness of one-layer PEI based on DFT calculation. (B) Infrared thermal image of the milling jar taken immediately after 5-h milling at 500 rpm.

According to equation $F = \rho \frac{\Delta V}{Y} A > P \times A$ (1) in the main text, with the lowest Y the graphene will experience the highest shear stress. To calculate the minimum viscosity required for graphene exfoliation, we used the most extreme scenario that there are only one-layer PEI molecules adsorbed on the surface of bilayer graphene. Based on DFT calculation (Supplementary Section 5.5), the thickness of one-layer PEI is around 0.6 nm (Fig. 5.11A) and the minimal required shear stress for sliding is in the zigzag direction locating at 0.116 Gpa. According to DEM simulation (Supplementary Section 5.2), the most probable relative velocity of grinding balls during operation (500 rpm) is at v=0.075 m/s. As a result, the required minimum viscosity is 1,005 mpa·s. However, the calculated minimum viscosity is significantly lower than the experimental results (between 7,235 and 16,858 mPa.s). It is worthy to note that the viscosity of PEI mixture was measured at room temperature (20 °C). To understand the deviation between theoretical calculation and experimental results, we measured the in-situ temperature of the milling jar using an infrared thermal camera right after 5 hours of exfoliation at 500 rpm. We found that the temperature of PEI mixture during exfoliation process was around 50 °C. As shown in Supplementary Table. 1.1, temperature has a significant impact on the viscosity of PEI mixture. As temperature increases from room temperature to 50 °C, the viscosity of PEI mixture declined around 7 times. This means that the viscosity of PEI, 7,235 and 16,858 mPa.s, which are measured at room temperature are only around 1,000 and 2,500 mPa.s,

respectively. The results indicate a reliable agreement between theoretical calculation and experimental results.

Supplementary Section 6. Universality of sticky mechanical exfoliation

Supplementary 6.1 Other layered crystals

Supplementary 6.1.1 Exfoliation of TAPB-PDA COF

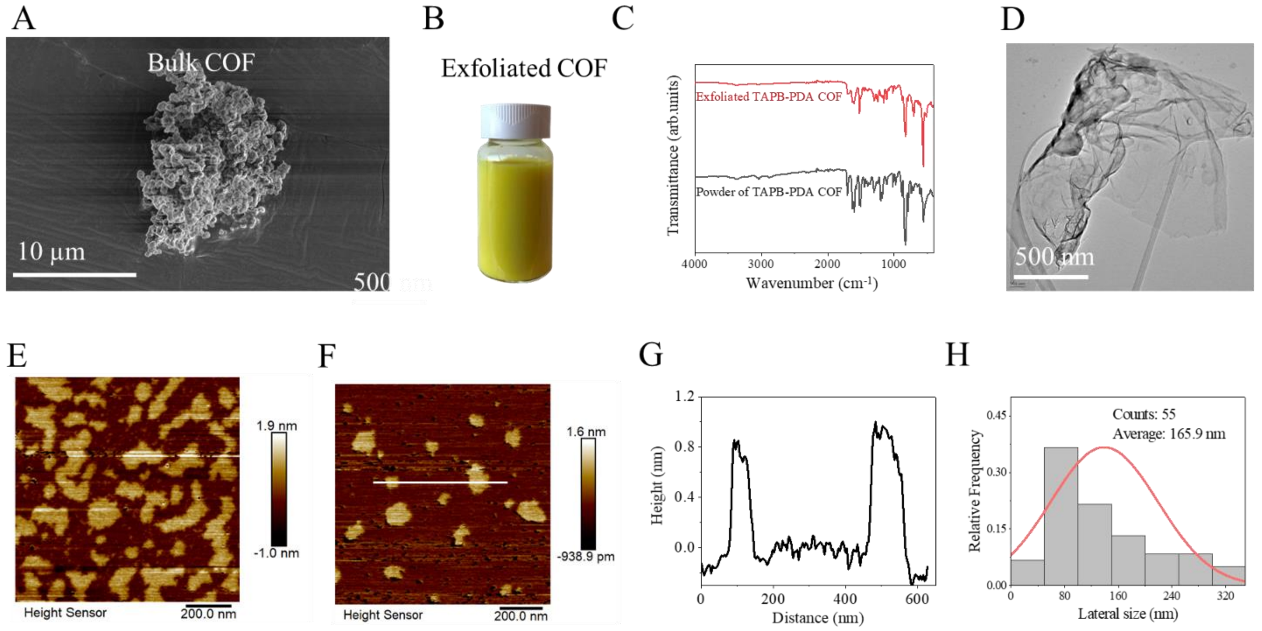

Supplementary Fig. 6.1. Characterization of the exfoliation TAPB-PDA COF by Sticky exfoliation. (A) SEM image of synthesized powder of bulk COF. (B) Photo of exfoliated COF nanosheets water dispersion at a concentration of 1 mg/mL. (C) FTIR patterns of bulk COF, and exfoliated COF. (D, E) Selected TEM images of exfoliated COF. (F) Size distribution of exfoliated COF based on TEM imaging. (G, H) Selected AFM image of exfoliated COF (J) Height profile of exfoliated COF based on G. Exfoliation conditions: Bulk=0.5g, PEI Vis-5=2g, Milling time=15h, Milling speed=500 rpm.

Exfoliation of porous covalent organic framework TAPB-PDA was also studied. Although the skeleton of TAPB-PDA COF is highly hydrophobic, the obtained COF nanosheets still can be dispersed in water and show as a yellow dispersion (Supplementary Fig. 6.1B). The Fourier-transform infrared (FT-IR) spectra of TAPB-PDA COF powder and exfoliated COF nanosheets are very similar, both of them display a sharp imine peak at around 1620, suggesting that the imine-bonded structure remains after exfoliation (Supplementary Fig. 6.1C). SEM and TEM images confirm that the lateral size of COF was downsized from several microns to hundreds of nanometers with an averaged lateral size of 165.9 (Supplementary Fig. 6.1H). AFM statics show that almost all the thickness of the

observed nanosheets locates around 1nm with deviations within 0.3nm, indicating an ultrahigh percentage of monolayers contained in the obtained products (Supplementary Figs. 6.1E-G).

Supplementary 6.1.2 Exfoliation of ZIF-L

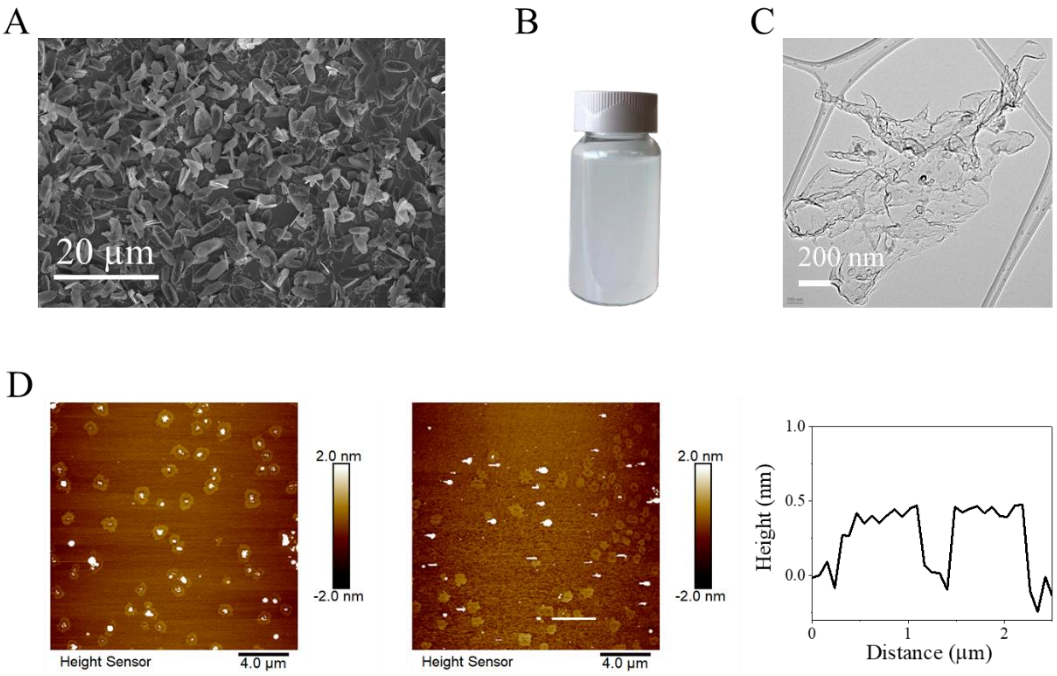

Supplementary Fig. 6.2. Characterization of the exfoliation TAPB-PDA COF by Sticky exfoliation. (A) SEM image of bulk ZIF-L powder. (B) Exfoliated ZIF-L nanosheets dispersed in ethanol at a concentration of 1 mg/mL. (C) selected TEM image of exfoliated ZIF-L nanosheet. (D) Selected AFM images of exfoliated ZIF-L nanosheets and the height profile along the white line. Exfoliation conditions: Bulk=0.5g, PEI Vis-5=2g, Milling time=5h, Milling speed=500 rpm.

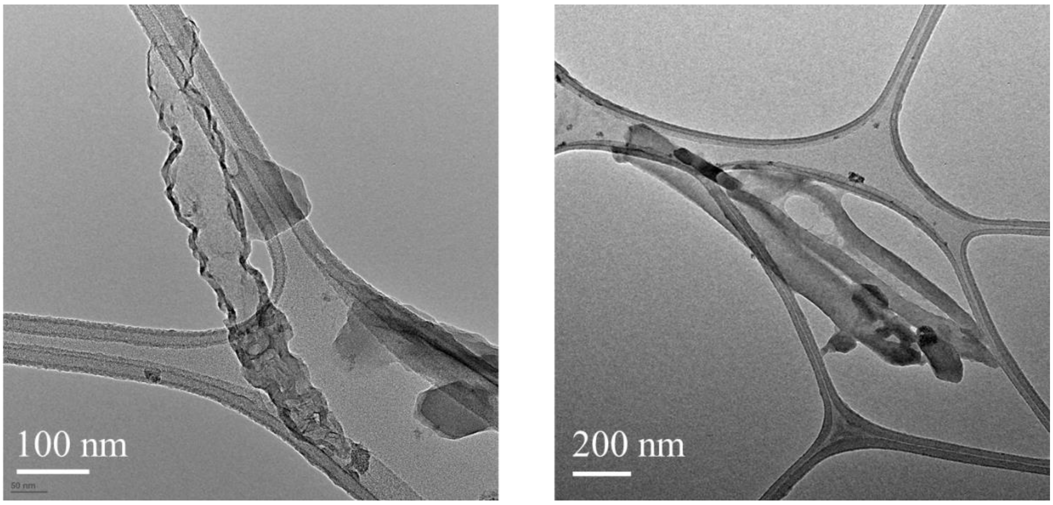

Supplementary Fig. 6.3. TEM imaging of the transformation of ZIF-L nanosheets to ZIF-8 particles. ZIF-L nanosheets in different stage were observed by TEM imaging. The left one is in the middle of transformation as it remains part of the nanosheet-like structure. The right one shows a tick-like structure, meaning it may have transformed into ZIF-8 particles.

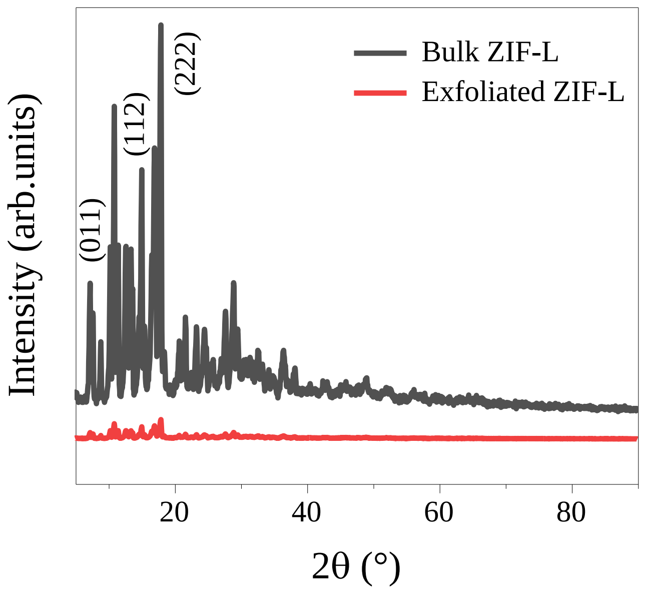

Supplementary Fig. 6.4. XRD patterns of bulk ZIF-L and exfoliated ZIF-L

SEM image of bulk ZIF-L confirm that the obtained powder is composed of numerous particles with leaf-like shapes. Each individual particle has a size of several microns and a thickness of around 100 nm, agreeing with the reported structure of ZIF-L[2]. The exfoliated ZIF-L can be dispersed in ethanol (Supplementary Fig. 6.2 B). We chose ethanol instead of water is under the consideration that ZIF-L is not stable in aqueous environment. TEM and AFM statics show that the lateral size of ZIF-L nanosheets reduces to around 1.2 μm (Supplementary Figs. 6.2 C-D). The thickness of these nanosheets is mostly at around 0.5 nm with deviations less than 0.2 nm (Supplementary Fig. 6.2 D).

However, ZIF-L nanosheets are not stable as they can easily transform to ZIF-8 particles in organic solvents or by heating according to previous research[5]. We also noted this transformation process from TEM images of two nanosheets in two different phase transformation status from a same sample (Supplementary Fig. 6.3). This transformation can be evident from XRD patterns of the exfoliated nanosheets as well, which shows the characteristic peaks at 2θ of 7.3°, 12.7°, and 18.0°, corresponding to the (011), (112) and (222) planes of the structure of ZIF-8 (Supplementary Fig. 6.4).

Supplementary 6.1.3 Exfoliation of g-C_3N_4

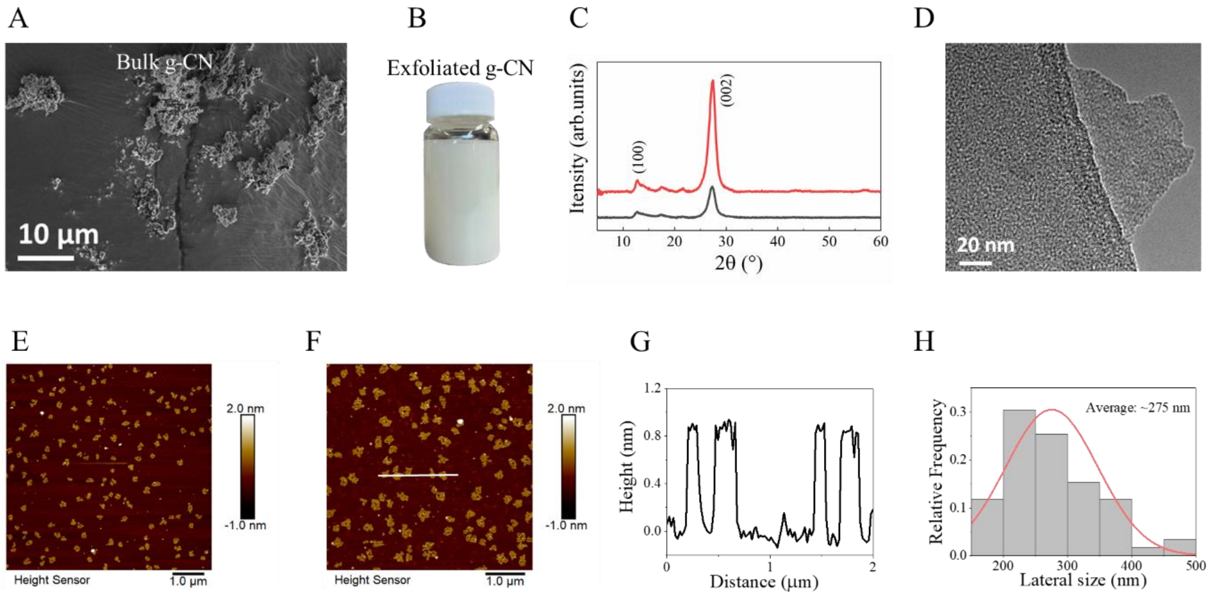

Supplementary Fig. 6.5. Characterization of the exfoliation g-C_3N_4 by Sticky exfoliation. (A) SEM image of Bulk g-C_3N_4. (B) Photo of exfoliated g-C_3N_4 water dispersion at a concentration of 1 mg/mL. (C) XRD patterns of bulk g-C_3N_4, and exfoliated g-C_3N_4. (D) Selected TEM image of exfoliated g-C_3N_4. (E, F) Selected AFM images of exfoliated g-C_3N_4. (G) Height profile along the white line in F. (H) Size distribution of exfoliated g-C_3N_4 based on E and F. Exfoliation conditions: Bulk=0.5g, PEI Vis-5=2g, Milling time=15h, Milling speed=500 rpm.

Exfoliation of g-C_3N_4 by sticky exfoliation method was also studied. The exfoliated g-C_3N_4 nanosheets can be finely dispersed in water similar to the case of graphene. Unlike graphene, g-C_3N_4 water dispersion presents as a light-yellow dispersion instead of black one. The XRD patterns of exfoliated g-C_3N_4 show the characteristic peaks including (002), (100), but these peaks are broader and with reduced intensities compared to that of bulk powder, indicating its reduced size and thickness. The SEM and TEM images confirm the lateral size reduces from several microns to hundreds of nanometers (averaged at 275 nm). AFM statics show that almost all the thickness of the observed nanosheets is less than 1nm with deviations less than 0.2 nm, indicating an ultrahigh percentage of monolayers contained in the obtained products.

In summary, the developed sticky milling is suitable for the exfoliation of TAPB-PDA COF, g-C_3N_4 and ZIF-L. The SEM, AFM and TEM images confirm that the lateral size and thickness of these layered bulk materials were downsized to hundreds of nanometers and thickness reduced to sub-1 nm region with deviations within 0.3 nm (Supplementary Figs. 6.1, 6.2, 6.5). The heights of these nanosheets display an ultra-narrow unimodal distribution, which is obviously different from previously observed normal distribution in terms of heights in other mechanical exfoliated products[41, 53]. This could be the sign that the thickness of most nanosheets has been adequately reduced and has

reached a limit. Furthermore, based on our experimental and simulation experience gained from the exfoliation of graphene and h-BN, the apparent heights of monolayered graphene locate in the range of 0.5 nm to 1 nm. Both the nanosheets and the residual PEI contribute to the observed apparent heights by AFM. We, therefore, take the nanosheets of g-C_3N_4, TAPB-PDA COF, ZIF-L with their thickness less than 1 nm as monolayers. From AFM statistics, these layered crystals can all be exfoliated into nanosheets with a high monolayer percentage of over 85%. The results consolidate the universality of this sticky exfoliation method for the production of monolayer nanosheets.

Supplementary 6.2 Exfoliation of BN

Supplementary Table 6.1. The milling parameter for BN

Sample name	PEI mixture	PEI loading amount (weight ratio to layered materials)	Milling speed (Revolution, rpm)	Milling time (hours)	Apparent yield (%)
Boron nitride-1 (BN-1)	Vis-5	2	500	15	61.7
Boron nitride-2 (BN-2)	Vis-5	2	500	25	75.9
Boron nitride-3 (BN-3)	Vis-5	2	600	15	82.62

Note: Considering that BN-2 needs long time operation and BN-3 requires high rotation speed, for the milling of BN-2 and BN-3, the mill stopped for 20 min after every 1-h running and changed its rotation direction in each turn. This can prevent the mill from overheating and provide efficient milling. The milling time in the table indicates the total running time. Pause time is not included.

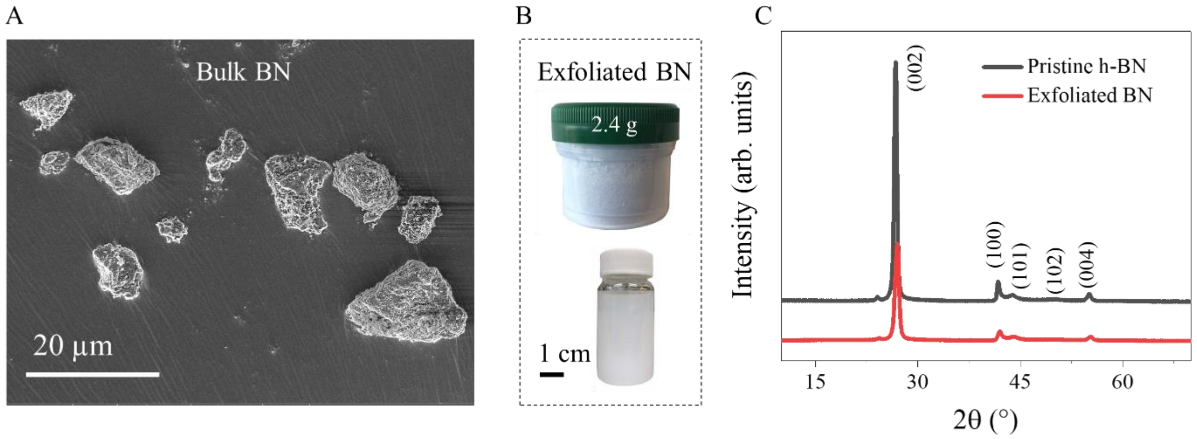

Supplementary Fig. 6.6. Exfoliation of h-BN. (A) SEM image of bulk BN particles. (B) Photographs of exfoliated BN-1 nanosheets in powder form and water dispersion at a concentration of 1 mg/mL. (C) XRD patterns of bulk h-BN and exfoliated BN-1 powder. Exfoliation conditions: PEI=Vis-5, Speed=500 rpm, BN=0.5g, PEI=1g, Milling time=15h.

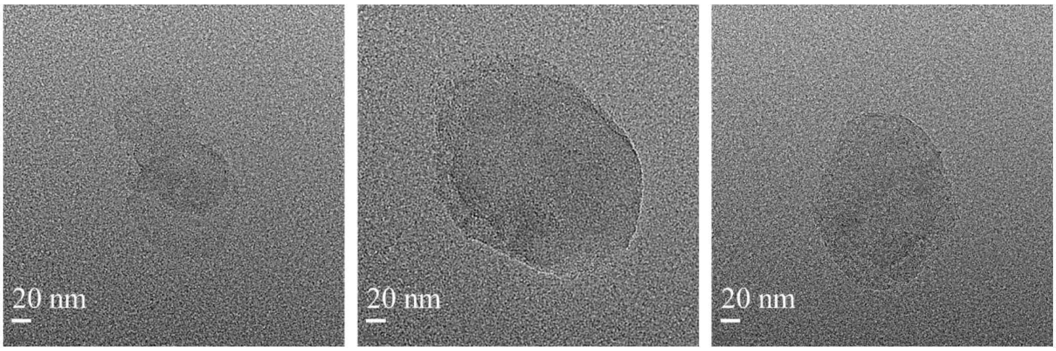

Supplementary Fig. 6.7. Exfoliation of h-BN. TEM images of BN-1 nanosheets being deposited on a carbon mesh. Exfoliation conditions: PEI=Vis-5, Speed=500 rpm, BN=0.5g, PEI=1g, Milling time=15h.

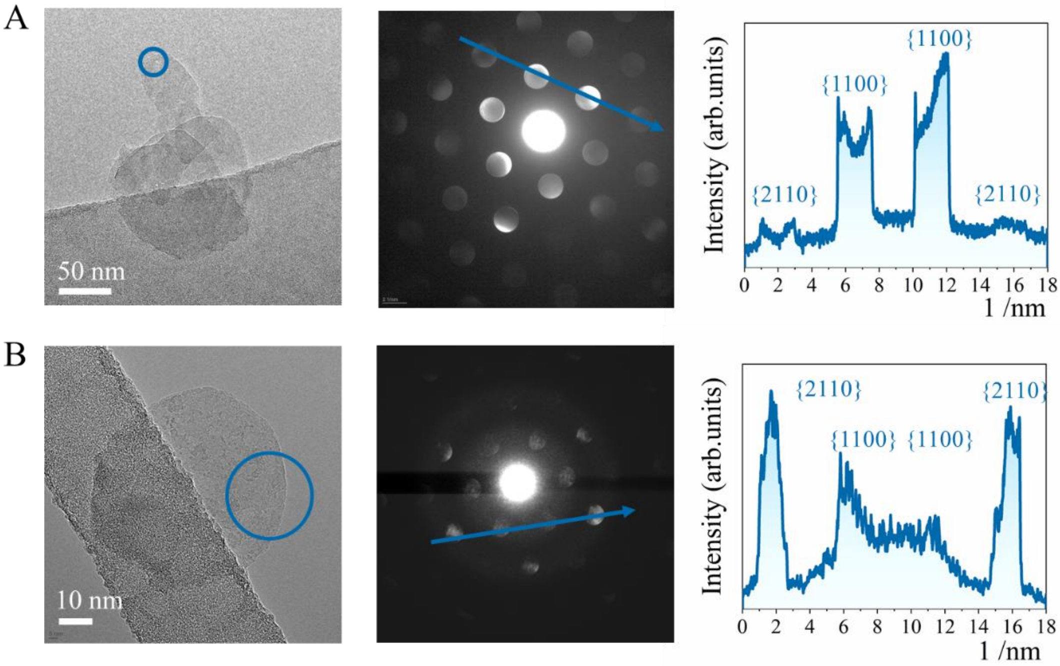

Supplementary Fig. 6.8. Exfoliation of h-BN. (A) (B) TEM images of BN-1 layers, the corresponding CBED pattern captured from the circled area, and the intensity of CBED spots along the lines. Exfoliation conditions: PEI=Vis-5, Speed=500 rpm, BN=0.5g, PEI=1g, Milling time=15h.

The adopted bulk BN is composed of particles in the range of several to tens of microns as indicated by SEM imaging (Supplementary Fig. 6.6A). At initial, we tried to keep the same milling parameters (PEI:h-BN=1:4, Speed=500 rpm, Milling time=15) as the case of graphite exfoliation. We found it was difficult to get a decent yield, often lower than 40%. To improve the exfoliation efficiency, we lowered the PEI addition to 2 times the bulk BN to enhance the shear stress. We found the apparent yield can be promoted to 61.7% through this strategy. Similar to graphene, the exfoliated BN can be finely dispersed in water, or processed into powder exhibiting fluffy texture (Supplementary Fig. 6.6B). The XRD patterns of bulk BN and exfoliated BN share the same peaks, indicating the largely preserved crystal structure (Supplementary Fig. 6.6C).

TEM, SEM and AFM statistics were applied to study the exfoliation efficiency. Unfortunately, AFM results show that an averaged thickness of h-BN nanosheets was as high as 7 nm and the percentage of monolayer nanosheets is only ~5% (Supplementary Fig. 6.10A). We assume that it could be the result of the much more intensive interlayer attraction force, and thus more difficult for exfoliation and stronger propensity to re-staking[74]. In order to understand how these multilayer h-BN nanosheets are formed, converged beam electric diffraction (CEBD) which focuses on a small area was performed to study these multilayer nanosheets. We found there seem to be two kinds of multilayer BN. One looks like that it is piled up by several pieces of BN nanosheets as shown in Supplementary Figure 6.8A. Although it shows as a multilayer BN, the diffraction pattern obtained on the edge of the nanosheet displays as a monolayer BN fingerprint (brighter inner spots than the outer spots). The results indicate that the observed BN sheet may consist of a piling of several monolayers. As a contrast, another kind of nanosheet that looks like an individual nanosheet but displays a typical diffraction pattern of multilayer BN with more intense outer spots than inner spots (Supplementary Fig 6.8B). This kind of multilayers may result from unsuccessful exfoliation. Therefore, we reckon that multilayer h-BN nanosheets may either come from restacking of nanosheets or those that have not been fully exfoliated.

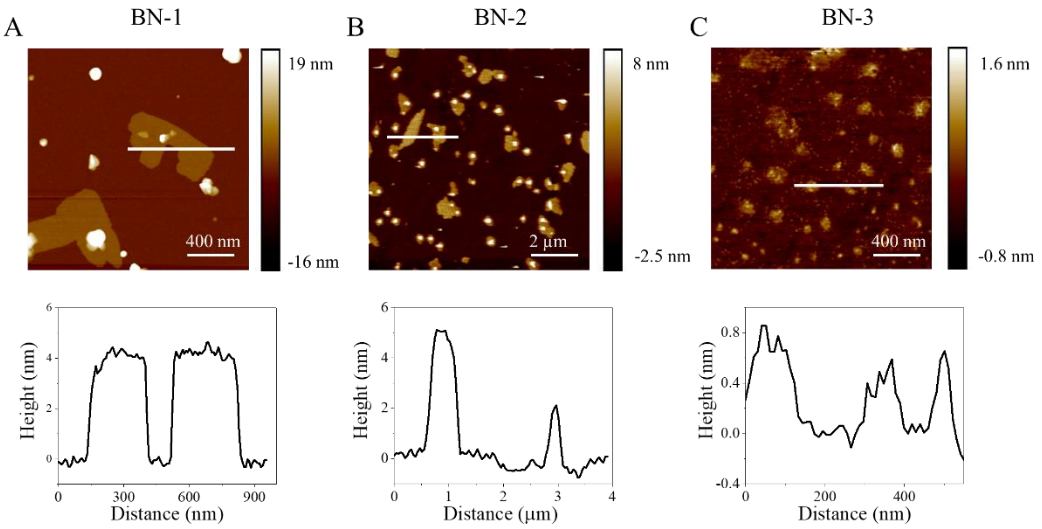

Supplementary Fig. 6.9. Exfoliation of h-BN. Selected AFM images and height profile along the white line of the obtained h-BN nanosheets under different conditions (A) BN-1, (B) BN-2, (C) BN-3. Experimental parameters of BN-1, 2, 3 are provided in Supplementary Table 6.1.

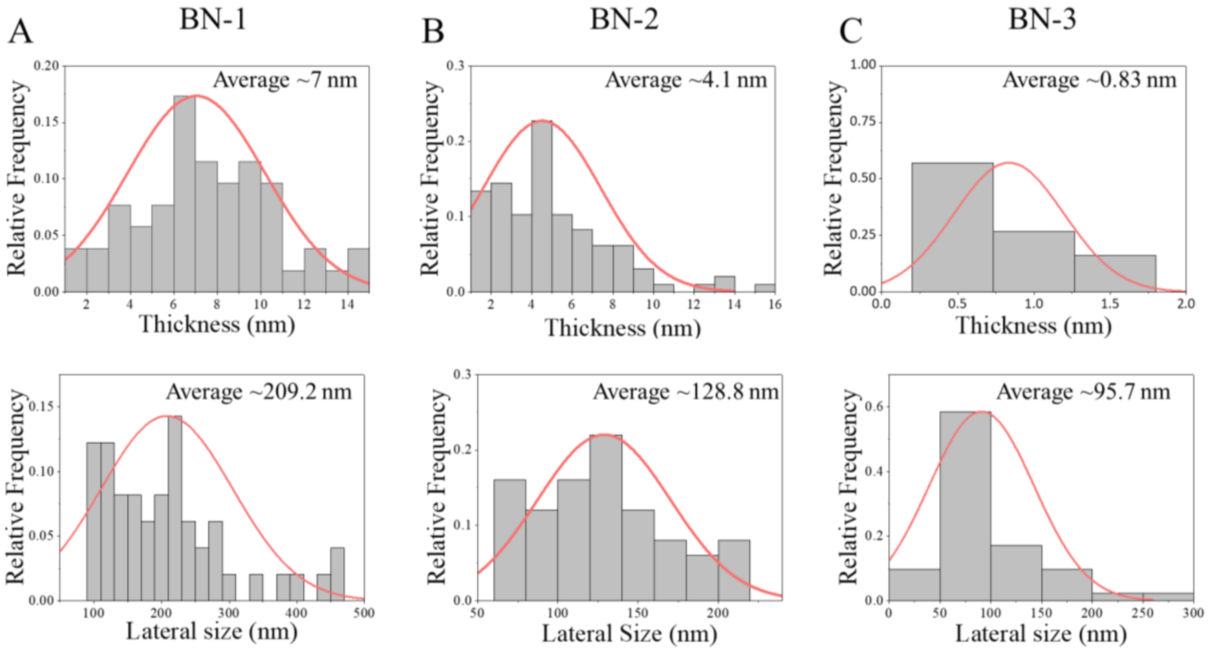

Supplementary Fig. 6.10. Exfoliation of h-BN. Statistical analysis of the obtained h-BN nanosheets in terms of their thickness and their lateral size based on AFM characterization. Experimental parameters of BN-1, 2, 3 are provided in Supplementary Table 6.1.

Based on our hypothesis about the cause of multilayer h-BN nanosheets, we tried to optimize experimental parameters to improve the monolayer percentage. Two strategies were exerted, one is raising milling time from 15h (BN-1) to 25h (BN-2), another one is increasing the milling speed from 500 rpm (BN-1) to 600 rpm (BN-3). Based on AFM statistical analysis towards over 50 pieces of exfoliated BN nanosheets, we found the average thickness reduced from 7 to 4.1 nm by prolonging the milling time, and further reduced to 0.83 nm by applying higher milling rotation speed (Supplementary Figs. 6.9-6.10). We also noted that the promoting of monolayer percentage is at the expense of losing lateral size much like the case observed in graphene. The average size fell from ~209.2 nm of BN-1 to ~95.7 nm of BN-3 (Supplementary Figs. 6.9-6.10).

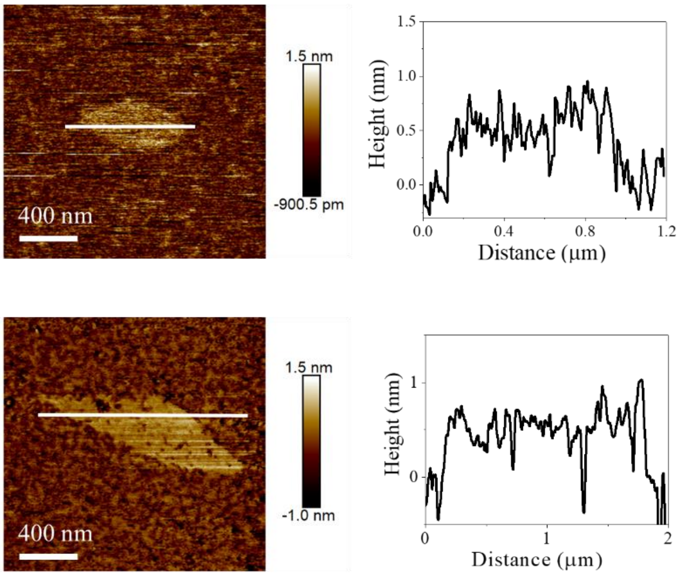

Supplementary Fig. 6.11. Selected AFM images and the height profiles of monolayer BN nanosheets.

AFM statistics show that the thinnest BN nanosheets hold heights around 0.6 nm (Figs. 6.11), similar to the heights of monolayer graphene. We believe that they are monolayer h-BN nanosheets because their apparent heights are less than the theoretical thickness of bilayer h-BN (~0.66 nm). To further confirm that the observed thinnest h-BN nanosheets are monolayered, we performed electric diffraction of the BN sample using a transmission electron microscope to find if there are any monolayers in the exfoliated products. Lager area pattern acquisition SAED instead of CBED is applied for the characterization of monolayered BN. A selected h-BN nanosheet displays a diffraction pattern corresponding to a typical fingerprint of monolayer BN with more intense inner spots ({1010} facets) than outer spots ({2110} facets) (Figs. 4g, h, main text)[41].

In summary, although bulk BN cannot be exfoliated to give a totally monolayered product, the monolayer percentage can be promoted from ~5% (BN-1) to ~14% (BN-2) by prolonging the milling time, and it can be further promoted to ~57% (BN-3) by applying higher milling rotation speed (Supplementary Figs. 6.10). This high monolayer percentage still shows superiority to other mechanical exfoliation methods as detailed in Supplementary Section 6.3.

Supplementary 6.3 Comparison with existing methods

Supplementary Table 6.2. Comparison with reported mechanical exfoliation methods in terms of monolayer percentage and yield.

Materials	Method	Thickness	Lateral Size	Monolayer percentage	Yield (%)	Reference
Boron nitride	Thermal oxidation etching + liquid exfoliation	<5 nm	~100 nm	Not discussed	-	52

	Hydroxide-Assisted Ball Milling	<10 layers, 3nm	1.5-2 μm	~5%	18	[75]
	Ball milling after Polydopamine (PDA)-coating	20 layers	20 μm	Not discussed	-	[76]
	Ball milling with urea	<2.5 nm	~100 nm	Not discussed	85	[55]
	Ball milling with sugar	4.4 nm	98 nm	~5%	98	[53]
	Compressible flow exfoliation	4.2 nm	276 nm	Not discussed	10	[77]
	Sonication with plant extracts	4.86 nm	77.7 nm	Not discussed	~23	[78]
	Sonication with PU precursor	~14.8 nm	~196.5 nm	<6%	-	[79]
	Supercritical fluid processing	100-200 nm	~0.5-1 μm	Not discussed	~10	[80]
	Intermediate-assisted grinding	4 nm	1.2 μm	~2%	67	[81]
	Sonication in solvent	-	100-5000 nm	<10%	0.2	[41]
	Bipolar electrochemistry	8.4 nm ± 3.3 nm	1.27 μm ±0.48 μm	Not discussed	-	[82]
	BN-1	7 nm	209.2 nm	~5%	61.7	This work
	BN-2	4.1 nm	128.8 nm	~13%	75.9	
	BN-3	0.83 nm	95.7 nm	~57%	82.6	
Carbon nitride	Ball milling with pyrenebutyric acid	1.5-20 nm	~138.7±0.7 nm	~7%	12.8	[56]
	Ball milling	0.5-0.7 nm	2-6 nm	~5%	15	[57]
	Sticky milling	0.8 nm	275 nm	~90%	98.4	This work
TAPB-PDA-COF	Sticky milling	0.8 nm	165.9 nm	~90%	42.4	This work
Zif-L	Sticky milling	0.5 nm	1.2 μm	~85%	38.7	This work

Note: There are hardly papers focusing on the monolayer percentage contained in their exfoliated products. For papers with thickness distribution data, we take the percentage of nanosheets with the thinnest thickness in the sub-nm region as the monolayer percentage in order to give a fair comparison.

Supplementary References

1. Zeng X*, et al.* Simultaneously tuning charge separation and oxygen reduction pathway on graphitic carbon nitride by polyethylenimine for boosted photocatalytic hydrogen peroxide production. *ACS Catal.* **10**, 3697-3706 (2020).

2. Chen R*, et al.* A two-dimensional zeolitic imidazolate framework with a cushion-shaped cavity for CO 2 adsorption. *Chem. Commun.* **49**, 9500-9502 (2013).

3. Matsumoto M*, et al.* Rapid, low temperature formation of imine-linked covalent organic frameworks catalyzed by metal triflates. *J. Am.n Chem. Soc.* **139**, 4999-5002 (2017).

4. Kang Y, Xia Y, Wang H, Zhang X. 2D laminar membranes for selective water and ion transport. *Adv. Funct. Mater.* **29**, 1902014 (2019).

5. Low Z-X*, et al.* Crystal transformation in zeolitic-imidazolate framework. *Crystal growth & design* **14**, 6589-6598 (2014).

6. Liu H, Xing Z, Zhang J, Pan JH, Wang H, Zhang X. A Facile Chemical-Free and Universal Method for Transfer of Ultrathin Graphene-Based Films. *Adv. Mater. Interfaces* **3**, 1600540 (2016).

7. Zhao C*, et al.* Layered nanocomposites by shear-flow-induced alignment of nanosheets. *Nature* **580**, 210-215 (2020).

8. Novoselov KS*, et al.* Two-dimensional atomic crystals. *PANS* **102**, 10451-10453 (2005).

9. Xu K, Cao P, Heath JR. Graphene visualizes the first water adlayers on mica at ambient conditions. *Science* **329**, 1188-1191 (2010).

10. May P, Khan U, O'Neill A, Coleman JN. Approaching the theoretical limit for reinforcing polymers with graphene. *J. Mater. Chem.* **22**, 1278-1282 (2012).

11. Hernandez Y*, et al.* High-yield production of graphene by liquid-phase exfoliation of graphite. *Nat. nanotechnol.* **3**, 563-568 (2008).

12. Silva DL*, et al.* Raman spectroscopy analysis of number of layers in mass-produced graphene flakes. *Carbon* **161**, 181-189 (2020).

13. Yuan S, Li Y, Xia Y, Selomulya C, Zhang X. Stable cation-controlled reduced graphene oxide membranes for improved NaCl rejection. *J. Membr. Sci.* **621**, 118995 (2021).

14. Paton KR*, et al.* Scalable production of large quantities of defect-free few-layer graphene by shear exfoliation in liquids. *Nat. Mater.* **13**, 624-630 (2014).

15. Soule D, Nezbeda C. Direct basal-plane shear in single-crystal graphite. *J. Appl. Phys.* **39**, 5122-5139 (1968).

16. Rooney A*, et al.* Anomalous twin boundaries in two dimensional materials. *Nat. Commun.* **9**, 1-7 (2018).

17. Li Z*, et al.* Mechanisms of liquid-phase exfoliation for the production of graphene. *ACS nano* **14**, 10976-10985 (2020).

18. Alzakia FI, Jonhson W, Ding J, Tan SC. Ultrafast Exfoliation of 2D Materials by Solvent Activation and One-Step Fabrication of All-2D-Material Photodetectors by Electrohydrodynamic Printing. *ACS Appl. Mater. Interfaces* **12**, 28840-28851 (2020).

19. Li X, Shen J, Wu C, Wu K. Ball-Mill-Exfoliated Graphene: Tunable Electrochemistry and Phenol Sensing. *Small* **15**, 1805567 (2019).

20. He P*, et al.* Urea-assisted aqueous exfoliation of graphite for obtaining high-quality graphene. *Chem. Comm.* **51**, 4651-4654 (2015).

21. Khan U, O'Neill A, Lotya M, De S, Coleman JN. High-concentration solvent exfoliation of graphene. *small* **6**, 864-871 (2010).

22. Karagiannidis PG*, et al.* Microfluidization of graphite and formulation of graphene-based conductive inks. *ACS nano* **11**, 2742-2755 (2017).

23. Chu T*, et al.* Cationic Hexagonal Boron Nitride, Graphene, and MoS2 Nanosheets Heteroassembled with Their Anionic Counterparts for Photocatalysis and Sodium-Ion Battery Applications. *ACS Appl. Nano Mater.* **3**, 5327-5334 (2020).

24. Bellani S*, et al.* Scalable production of graphene inks via wet-jet milling exfoliation for screen-printed micro-supercapacitors. *Adv. Funct. Mater.* **29**, 1807659 (2019).

25. Zhao H, Xu B, Ding J, Wang Z, Yu H. Natural amino acids: high-efficiency intercalants for graphene exfoliation. *ACS Sustain. Chem. & Eng.* **7**, 18819-18825 (2019).

26. Ahmed H*, et al.* Ultrafast acoustofluidic exfoliation of stratified crystals. *Adv. Mater.* **30**, 1704756 (2018).

27. González-Domínguez JM, León V, Lucío MI, Prato M, Vázquez E. Production of ready-to-use few-layer graphene in aqueous suspensions. *Nat. protoc.* **13**, 495 (2018).

28. Leon V, Rodriguez AM, Prieto P, Prato M, Vazquez E. Exfoliation of graphite with triazine derivatives under ball-milling conditions: preparation of few-layer graphene via selective noncovalent interactions. *ACS nano* **8**, 563-571 (2014).

29. Buzaglo M, Bar IP, Varenik M, Shunak L, Pevzner S, Regev O. Graphite-to-Graphene: Total Conversion. *Adv. Mater.* **29**, 1603528 (2017).

30. Rubio N*, et al.* Production of water-soluble few-layer graphene mesosheets by dry milling with hydrophobic drug. *Langmuir* **30**, 14999-15008 (2014).

31. Arao Y*, et al.* Mass production of low-boiling point solvent-and water-soluble graphene by simple salt-assisted ball milling. *Nanoscale Adv.* **1**, 4955-4964 (2019).

32. González VJ*, et al.* Sweet graphene: exfoliation of graphite and preparation of glucose-graphene cocrystals through mechanochemical treatments. *Green Chem.* **20**, 3581-3592 (2018).

33. Chen S*, et al.* Simultaneous production and functionalization of boron nitride nanosheets by sugar-assisted mechanochemical exfoliation. *Adv. Mater.* **31**, 1804810 (2019).

34. Sierra U*, et al.* Coke-derived few layer graphene-like materials by mild planetary milling exfoliation. *Fuel* **262**, 116455 (2020).

35. Jeon I-Y*, et al.* Edge-carboxylated graphene nanosheets via ball milling. *PNAS* **109**, 5588-5593 (2012).

36. Ding J, Zhao H, Yu H. Graphene nanofluids based on one-step exfoliation and edge-functionalization. *Carbon* **171**, 29-35 (2021).

37. Cheng Z-L, Kong Y-C, Liu Z. Li+/Na+ Co-assisted hydrothermal exfoliation for graphite into few-layer graphene nanosheets and their excellent friction-reducing performance. *ACS Sustain. Chem. Eng.* **7**, 19770-19778 (2019).

38. Xia ZY*, et al.* The exfoliation of graphene in liquids by electrochemical, chemical, and sonication-assisted techniques: A nanoscale study. *Adv. Funct. Mater.* **23**, 4684-4693 (2013).

39. Luong DX*, et al.* Gram-scale bottom-up flash graphene synthesis. *Nature* **577**, 647-651 (2020).

40. Ren W, Cheng H-M. The global growth of graphene. *Nat. Nanotechnol.* **9**, 726-730 (2014).

41. Coleman JN*, et al.* Two-dimensional nanosheets produced by liquid exfoliation of layered materials. *Science* **331**, 568-571 (2011).

42. Jeon I-Y*, et al.* Large-scale production of edge-selectively functionalized graphene nanoplatelets via ball milling and their use as metal-free electrocatalysts for oxygen reduction reaction. *J. Am. Chem. Soc.* **135**, 1386-1393 (2013).

43. Shepherd E, Kitchener J. 474. The ionization of ethyleneimine and polyethyleneimine. *J. Am. Chem. Soc.*, 2448-2452 (1956).

44. Fadeeva V, Tikhova V, Nikulicheva O. Elemental analysis of organic compounds with the use of automated CHNS analyzers. *J. Anal. Chem.* **63**, 1094-1106 (2008).

45. Seah M. The quantitative analysis of surfaces by XPS: a review. *Surf. Interface Anal.* **2**, 222-239 (1980).

46. Beccat P, Da Silva P, Huiban Y, Kasztelan S. Quantitative surface analysis by XPS: application to hydro-treating catalysts; Analyse quantitative de surface par XPS (X-ray photoelectron spectroscopy): application aux catalyseurs d'hydrotraitement. *Oil & Gas Sci. Techno.* **54**, (1999).

47. Li D, Müller MB, Gilje S, Kaner RB, Wallace GG. Processable aqueous dispersions of graphene nanosheets. *Nat. Nanotechnol.* **3**, 101-105 (2008).

48. Chen H, Müller MB, Gilmore KJ, Wallace GG, Li D. Mechanically strong, electrically conductive, and biocompatible graphene paper. *Adv. Mater.* **20**, 3557-3561 (2008).

49. Wang X, Zhi L, Müllen K. Transparent, Conductive Graphene Electrodes for Dye-Sensitized Solar Cells. *Nano Lett.* **8**, 323-327 (2008).

50. Zhao W, Fang M, Wu F, Wu H, Wang L, Chen G. Preparation of graphene by exfoliation of graphite using wet ball milling. *J. Mater. Chem.* **20**, 5817-5819 (2010).

51. Zhu L, Zhao X, Li Y, Yu X, Li C, Zhang Q. High-quality production of graphene by liquid-phase exfoliation of expanded graphite. *Mater. Chem. Phys.* **137**, 984-990 (2013).

52. Ji X*, et al.* A novel top-down synthesis of ultrathin 2D boron nanosheets for multimodal imaging-guided cancer therapy. *Adv. Mater.* **30**, 1803031 (2018).

53. Gao S*, et al.* Improving the Acidic Stability of Zeolitic Imidazolate Frameworks by Biofunctional Molecules. *Chem* **5**, 1597-1608 (2019).

54. Ding J-H, Zhao H-R, Yu H-B. High-yield synthesis of extremely high concentrated and few-layered boron nitride nanosheet dispersions. *2D Materials* **5**, 045015 (2018).

55. Lei W, Mochalin VN, Liu D, Qin S, Gogotsi Y, Chen Y. Boron nitride colloidal solutions, ultralight aerogels and freestanding membranes through one-step exfoliation and functionalization. *Nat. Commun.* **6**, 1-8 (2015).

56. Ji J*, et al.* Simultaneous noncovalent modification and exfoliation of 2D carbon nitride for enhanced electrochemiluminescent biosensing. *J. Am. Chem. Soc.* **139**, 11698-11701 (2017).

57. Han Q*, et al.* Facile production of ultrathin graphitic carbon nitride nanoplatelets for efficient visible-light water splitting. *Nano Res.* **8**, 1718-1728 (2015).

58. Dong L, *et al.* A non-dispersion strategy for large-scale production of ultra-high concentration graphene slurries in water. *Nat. Commun.* **9**, 1-8 (2018).

59. Andreeva DV, *et al.* Two-dimensional adaptive membranes with programmable water and ionic channels. *Nat. Nanotechnol.* **16**, 174-180 (2021).

60. Zhang Z, *et al.* Bioinspired graphene oxide membranes with pH-responsive nanochannels for high-performance nanofiltration. *ACS nano* **15**, 13178-13187 (2021).

61. Cundall PA, Strack ODL. A discrete numerical model for granular assemblies. *Géotechnique* **29**, 47-65 (1979).

62. Hou QF, Dong KJ, Yu AB. DEM study of the flow of cohesive particles in a screw feeder. *Powder Technol.* **256**, 529-539 (2014).

63. Li X, Hou QF, Dong KJ, Zou RP, Yu AB. Promote cohesive solid flow in a screw feeder with new screw designs. *Powder Technol.y* **361**, 248-257 (2020).

64. Liu PY, Yang RY, Yu AB. Self-diffusion of wet particles in rotating drums. *Phys. Fluids* **25**, 063301-063312 (2013).

65. Zhu HP, Zhou ZY, Yang RY, Yu AB. Discrete particle simulation of particulate systems: Theoretical developments. *Chem. Eng. Sci.* **62**, 3378-3396 (2007).

66. Dong KJ, Yu AB, Brake I. DEM simulation of particle flow on a multi-deck banana screen. *Miner. Eng.* **22**, 910-920 (2009).

67. Kresse G, Furthmüller J. Efficiency of ab-initio total energy calculations for metals and semiconductors using a plane-wave basis set. *Comput. mater. sci.* **6**, 15-50 (1996).

68. Kresse G, Furthmüller J. Efficient iterative schemes for ab initio total-energy calculations using a plane-wave basis set. *Phy. Rev. B* **54**, 11169 (1996).

69. Perdew JP, Burke K, Ernzerhof M. Generalized gradient approximation made simple. *Phys. Rev. Lett.* **77**, 3865 (1996).

70. Blöchl PE. Projector augmented-wave method. *Phy. Rev. B* **50**, 17953 (1994).

71. Grimme S, Antony J, Ehrlich S, Krieg H. A consistent and accurate ab initio parametrization of density functional dispersion correction (DFT-D) for the 94 elements H-Pu. *Chem. Phys.* **132**, 154104 (2010).

72. Liu Z, Zhang S-M, Yang J-R, Liu JZ, Yang Y-L, Zheng Q-S. Interlayer shear strength of single crystalline graphite. *Acta Mech. Sin.* **28**, 978-982 (2012).

73. Donnet C, Martin J, Le Mogne T, Belin M. Super-low friction of MoS2 coatings in various environments. *Tribol. Int.* **29**, 123-128 (1996).

74. Jung JH, Park C-H, Ihm J. A rigorous method of calculating exfoliation energies from first principles. *Nano Lett.* **18**, 2759-2765 (2018).

75. Lee D, Lee B, Park KH, Ryu HJ, Jeon S, Hong SH. Scalable exfoliation process for highly soluble boron nitride nanoplatelets by hydroxide-assisted ball milling. *Nano Lett.* **15**, 1238-1244 (2015).

76. Wu H, Zhao W, Hu H, Chen G. One-step in situ ball milling synthesis of polymer-functionalized graphene nanocomposites. *J. Mater. Chem.* **21**, 8626-8632 (2011).

77. Rizvi R*, et al.* High-throughput continuous production of shear-exfoliated 2D layered materials using compressible flows. *Adv. Mater.* **30**, 1800200 (2018).

78. Deshmukh AR, Jeong JW, Lee SJ, Park GU, Kim BS. Ultrasound-assisted facile green synthesis of hexagonal boron nitride nanosheets and their applications. *ACS Sustain. Chem. Eng.* **7**, 17114-17125 (2019).

79. Zhu X*, et al.* Hexagonal Boron Nitride–Enhanced Optically Transparent Polymer Dielectric Inks for Printable Electronics. *Adv. Funct. Mater.* **30**, 2002339 (2020).

80. Thangasamy P, Sathish M. Supercritical fluid processing: a rapid, one-pot exfoliation process for the production of surfactant-free hexagonal boron nitride nanosheets. *CrystEngComm* **17**, 5895-5899 (2015).

81. Zhang C*, et al.* Mass production of 2D materials by intermediate-assisted grinding exfoliation. *Natl. Sci. Rev.* **7**, 324-332 (2020).

82. Wang Y, Mayorga-Martinez CC, Chia X, Sofer Z, Pumera M. Nonconductive layered hexagonal boron nitride exfoliation by bipolar electrochemistry. *Nanoscale* **10**, 7298-7303 (2018).
